# Supplementary material for: pH-gated nanoparticles selectively regulate lysosomal function of tumour-associated macrophages for cancer immunotherapy
Source: Nat Commun. 2023 Sep 21;14:5888. doi: 10.1038/s41467-023-41592-0 (PMC10514266; doi:10.1038/s41467-023-41592-0)
Supplement: Supplementary file 1 — Supplementary Information [file 41467_2023_41592_MOESM1_ESM.pdf]

pH-gated nanoparticles selectively regulate lysosomal function of tumour-associated macrophages for cancer immunotherapy

*Mingmei Tang[1,2], Binlong Chen[2], Heming Xia[2], Meijie Pan[2], Ruiyang Zhao[2], Jiayi Zhou[2], Qingqing Yin[2], Fangjie Wan[2], Yue Yan[2], Chuanxun Fu[2], Lijun Zhong[3], Qiang Zhang[1,2], Yiguang Wang[1,2,4]**

[1]State Key Laboratory of Natural and Biomimetic Drugs, School of Pharmaceutical Sciences, Peking University, Beijing, China. [2]Beijing Key Laboratory of Molecular Pharmaceutics and New Drug Delivery System, School of Pharmaceutical Sciences, Peking University, Beijing, China. [3]Center of Medical and Health Analysis, Peking University Health Science Center, Beijing, China. [4]Chemical Biology Center, Peking University, Beijing, China.

* Corresponding author.
E-mail address: yiguang.wang@pku.edu.cn

Table of contents

1. Supplementary figures 4

Supplementary Fig. 1. Polarization of macrophages to different phenotypes. 4

Supplementary Fig. 2. Lysosomal pH measurement of BMDMs. 5

Supplementary Fig. 3. Lysosomal pH measurement of different cell lines. 6

Supplementary Fig. 4. The synthetic route of PEG-*b*-P(R_1-*r*-R_2-AMA) block copolymers and probe conjugation. 7

Supplementary Fig. 5. Characterization of pH-dependent fluorescence spectra of PGNs library. 8

Supplementary Fig. 6. Characterization of $PGN_{4.9}$ nanoparticle labelled with visible-light fluorophores pairs. 9

Supplementary Fig. 7. Intracellular distribution of $PGN_{4.9}$ on BMDMs after incubation for 2 h. 10

Supplementary Fig. 8. CLSM images of BMDMs treated with PGN library using a pulse-chase assay. 11

Supplementary Fig. 9. Representative confocal images of different cell lines incubated with $PGN_{6.3}$ via a pulse-chase assay. 12

Supplementary Fig. 10. Representative confocal images of different cell lines incubated with $PGN_{4.9}$ via a pulse-chase assay. 13

Supplementary Fig. 11. Representative confocal images of different cell lines incubated with $PGN_{4.3}$ via a pulse-chase assay. 14

Supplementary Fig. 12. Flow cytometry of BMDMs incubated with $PGN_{4.9}$ in vitro. 15

Supplementary Fig. 13. Confocal images of endocytic organelles distribution of $PGN_{4.9}$ nanoprobe in 4T1 tumour-bearing mice. 16

Supplementary Fig. 14. Characterization of PGN nanoparticles labeled with near-infrared fluorophores pairs. 17

Supplementary Fig. 15. In vivo long-term monitoring of PGNs distribution in orthotopic 4T1 breast tumour xenografts. 18

Supplementary Fig. 16. Real-time monitoring of PGNs activation in CT26 colorectal tumour model. 19

Supplementary Fig. 17. Accumulation level and activation efficiency 20

Supplementary Fig. 18. Microscopic distribution of PGNs. 21

Supplementary Fig. 19. Ratiometric signals as a function of M2-like macrophage content in different tumour models. 22

Supplementary Fig. 20. The effect of macrophage depletion on $PGN_{4.9}$ activation in 4T1 tumour-bearing mice. 23

Supplementary Fig. 21. Biodistribution of PGNs in lymph nodes from 4T1 tumour-bearing mice. 24

Supplementary Fig. 22. Synthetic routes of AND-gate drug-conjugated copolymers. 25

Supplementary Fig. 23. ^{1}H-NMR spectra 26

Supplementary Fig. 24. Characterization of $PGN_{4.9}$ and NPGN nanoadjuvants in vitro. 27

Supplementary Fig. 25. Proinflammatory cytokines and acute systemic toxicity analysis. 28

Supplementary Fig. 26. Representative histological features 29
Supplementary Fig. 27. Changes of morphological phenotype in BMDMs 30
Supplementary Fig. 28. Repolarization of M2-like macrophage to M1-like phenotype by $PGN_{4.9}$ in vitro. 31
Supplementary Fig. 29. Confocal images of pretreated BMDMs incubated with $PGN_{4.9}$ nanoreporters. 32
Supplementary Fig. 30. The changes of lysosomal pH in M2-like BMDMs treated with IMDQ formulations. ... 33
Supplementary Fig. 31. Evaluation of protein degradation assays. 34
Supplementary Fig. 32. Up-regulation of costimulators in BMDMs for antigen presentation. 35
Supplementary Fig. 33. The expression of MHC-I molecules on BMDMs. 36
Supplementary Fig. 34. Effect of various IMDQ formulations on antigen cross-presentation. 37
Supplementary Fig. 35. Repolarization of M2-like macrophages to M1 phenotype. 38
Supplementary Fig. 36. Repolarization of M2-like macrophages to M1 phenotype in vivo. 39
Supplementary Fig. 37. Quantification of tumour infiltration for different immune cells. 40
Supplementary Fig. 38. $PGN_{4.9}$ promotes antigen processing and presentation in vivo. 41
Supplementary Fig. 39. Long-term immune memory. 42
Supplementary Fig. 40. Immunotherapy efficacy on 4T1-luc breast cancer. 43
Supplementary Fig. 41. Anti-metastasis study. 44
Supplementary Fig. 42. In vivo depletion of macrophages in 4T1 breast tumour model. 45
Supplementary Fig. 43. In vivo depletion of T cells in 4T1 breast tumour model. 46
Supplementary Fig. 44. Combined immunotherapy of $PGN_{4.9}$ and PDPA-DTX. 47
Supplementary Fig. 45. In vivo therapeutic efficacy of $PGN_{4.9}$ in MC38 colorectal tumour model. 48
Supplementary Fig. 46. Combined immunotherapy of $PGN_{4.9}$ and α-PD1. 49
2. Supplementary Tables 50
Supplementary Table 1. Characterization of synthetic copolymers and fluorescent nanoparticles 50
Supplementary Table 2. Characterization of polymer-drug conjugates 51

1. Supplementary figures

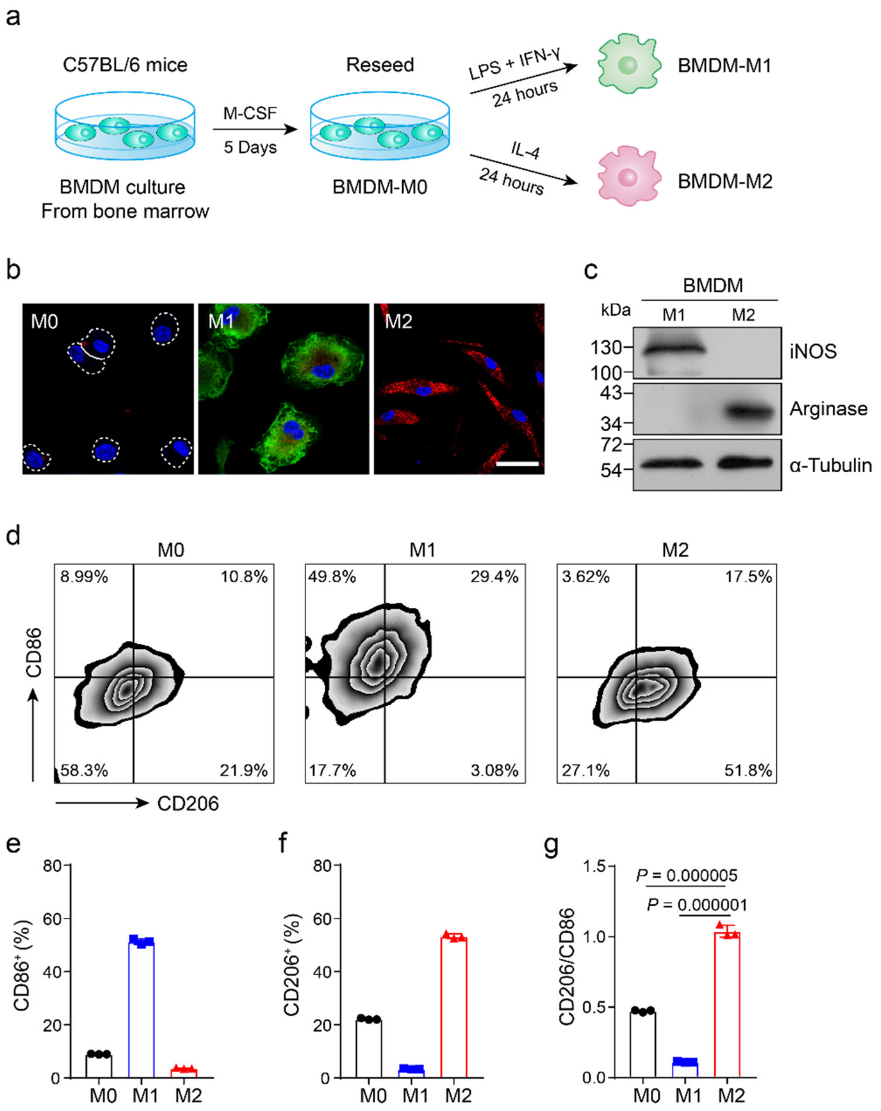

Supplementary Fig. 1. Polarization of macrophages to different phenotypes. (a) Schematic overview of macrophage differentiation in vitro. **(b)** Confocal images of macrophages with different phenotypes stained for nucleus (blue), iNOS (M1-like macrophage marker, green), and CD206 (M2-like macrophage marker, red) (*n* = 3 experiments). Scale bar, 20 μm. **(c)** Western blot analyses of iNOS and Arginase (M2-like macrophage marker) expression (*n* = 3 experiments). **(d)** Representative flow cytometric plots of $CD86^+$ $CD206^-$ M1-like macrophages and $CD86^-$ $CD206^+$ M2-like macrophages after incubating with different cytokines. **(e, f)** Quantitative polarization efficiency to M1- or M2-like phenotypes. **(g)** The ratio of CD86 and CD206 expression for different phenotypic macrophages (*n* = 3 experiments, one-way ANOVA followed by Tukey's multiple comparisons test). Source data are provided as a Source Data file.

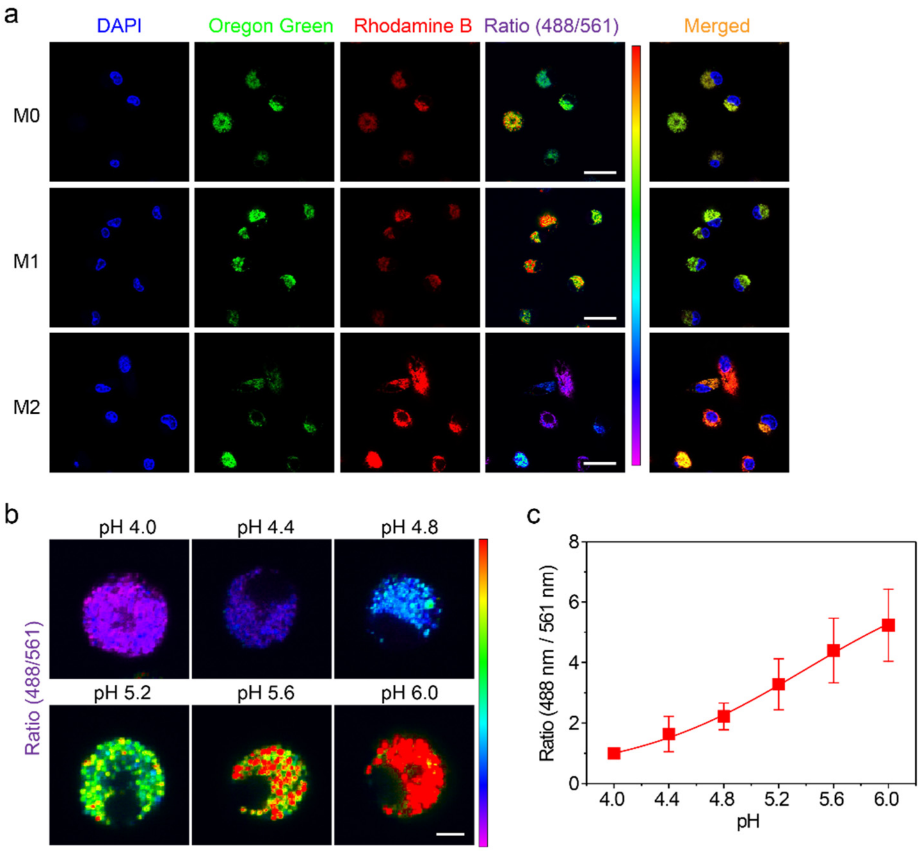

Supplementary Fig. 2. Lysosomal pH measurement of BMDMs. (a) Confocal images of BMDMs with different phenotypes incubated with fluorescent dextran cocktail via a pulse-chase assay. Blue, nucleus; green, Oregon Green; red, Rhodamine B; fire, ratio of green versus red signals. Scale bar, 50 μm. **(b)** Representative ratiometric images of BMDMs treated with nigericin in buffer solutions with different pH (n = 3 experiments). **(c)** The fitting standard curve of lysosomal pH value in BMDMs derived from C57BL/6 mice (n = 10 cells). Scale bar, 10 μm.

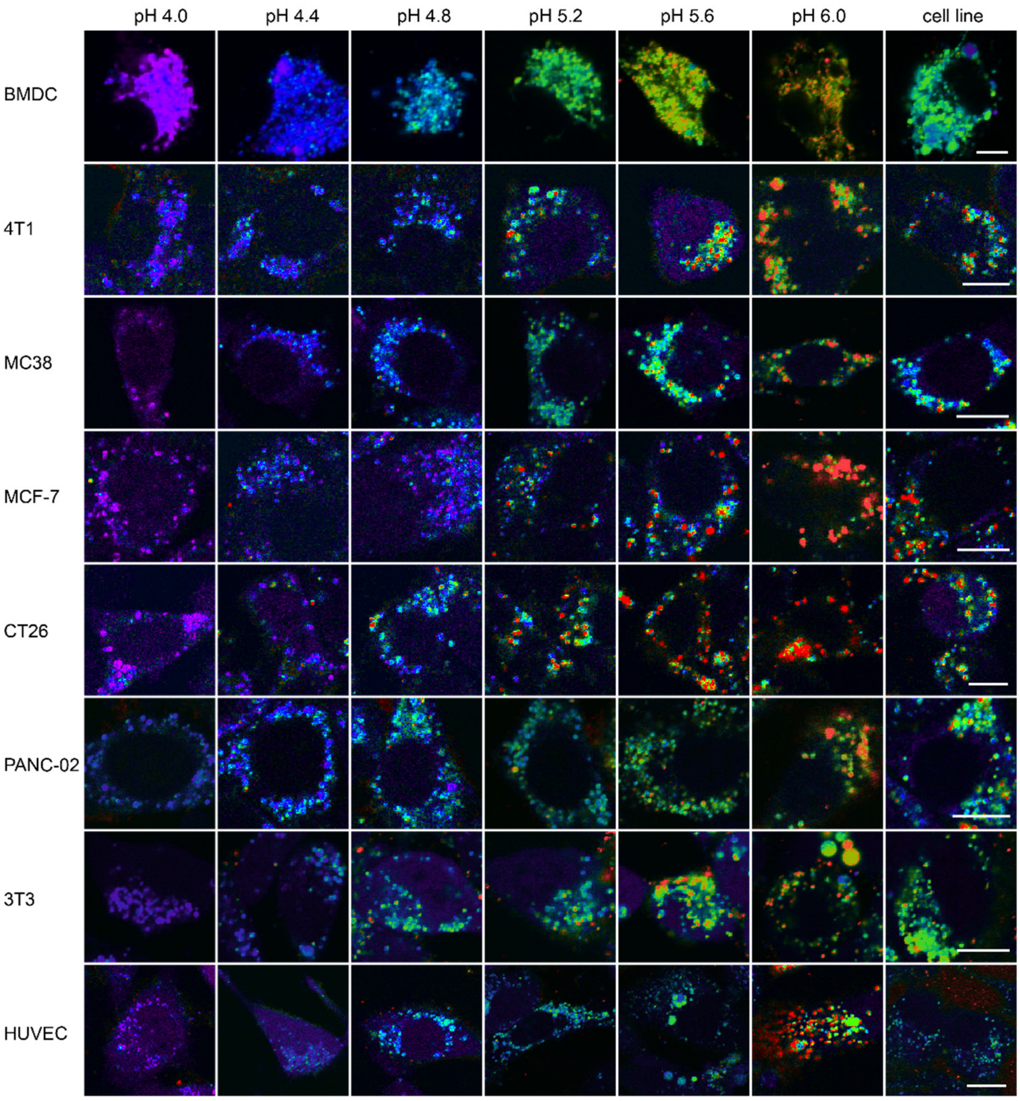

Supplementary Fig. 3. Lysosomal pH measurement of different cell lines. Ratiometric images of different cell lines in buffer solutions with different pH and the corresponding cells in culture medium (*n* = 3 experiments). Scale bar, 10 μm.

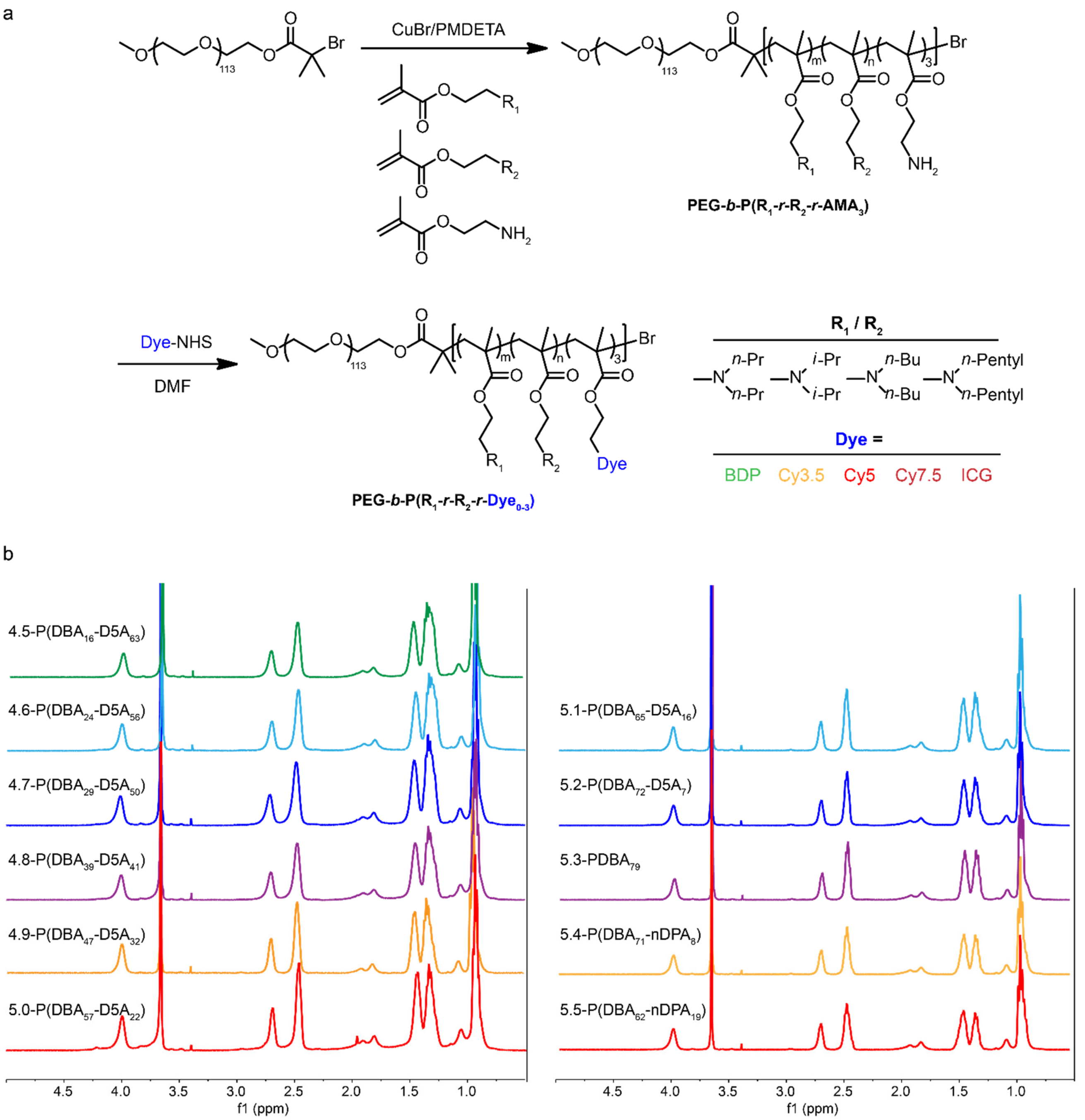

Supplementary Fig. 4. The synthetic route of PEG-*b*-P(R_1-*r*-R_2-AMA) block copolymers and probe conjugation. (a) The pH transition (pH_t) of PGNs library can be tuned by adjusting the molar fractions of the two hydrophobic monomers. **(b)** ^{1}H-NMR spectra of PEG-*b*-P(R_1-*r*-R_2-AMA_3) copolymer compositions with different pH_t values. The monomer compositions of P(DBA-*r*-D5A) and P(DBA-*r*-nDPA) copolymers were calculated from the peaks at 1.3 ppm and 1.4 ppm.

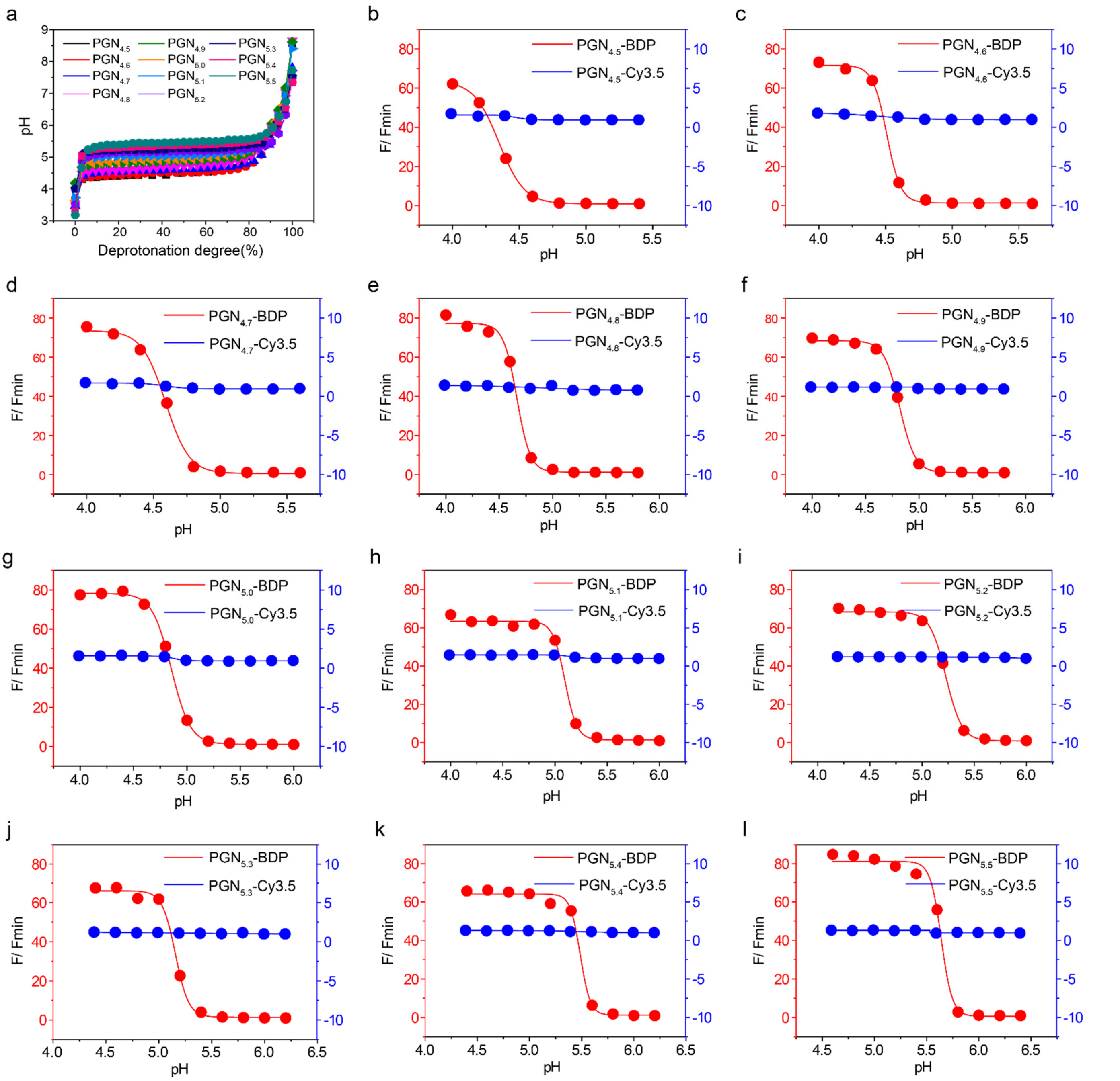

Supplementary Fig. 5. Characterization of pH-dependent fluorescence spectra of PGNs library. (a) pH titration and pK_a measurements of PGNs library. **(b-l)** BDP- and Cy3.5-conjugated PGN nanoreporters with pH transition (pH_t) from 4.5 to 5.5. Fluorescence intensity of BDP (red) and Cy3.5 (blue) as a function of pH for PGNs library. F is the fluorescence intensity of PGNs at any given pH value, and F_{min} represents the minimal fluorescence intensity at OFF state, respectively (*n* = 1 experiment).

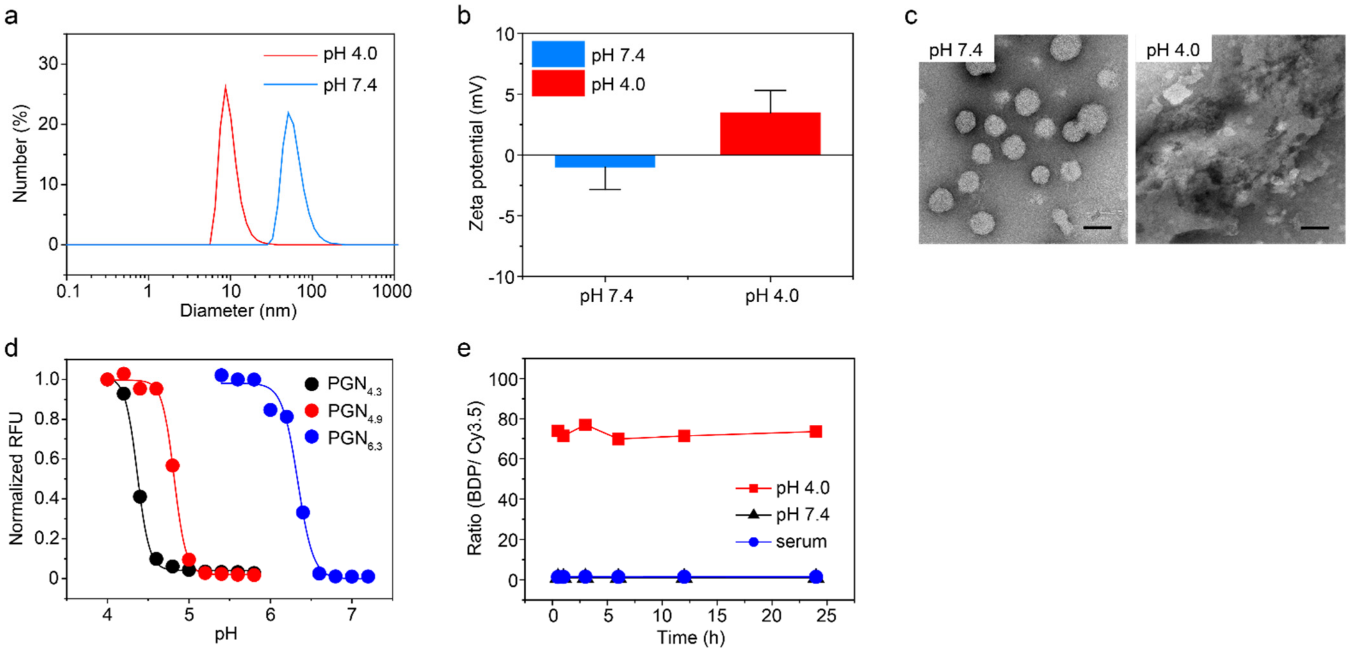

Supplementary Fig. 6. Characterization of $PGN_{4.9}$ nanoparticle labelled with visible-light fluorophores pairs. (a) Particle size distribution and **(b)** zeta potentials of $PGN_{4.9}$ nanoparticles (100 µg mL^{-1}) at pH 4.0 and 7.4 (n = 3 experiments). **(c)** TEM images of $PGN_{4.9}$ (800 µg mL^{-1}) at pH 4.0 and 7.4 (n = 3 experiments). Scale bar, 100 nm. **(d)** Fluorescence activation ratio as the function of pH for PGN-BDP/Cy3.5 nanoparticles with different pH_t (n = 1 experiment). **(e)** The fluorescence intensity of $PGN_{4.9}$-BDP/Cy3.5 nanoparticles (100 µg mL^{-1}) in PBS buffer with pH 7.4 or 4.0 and fresh serum at specific time-points (n = 1 experiment).

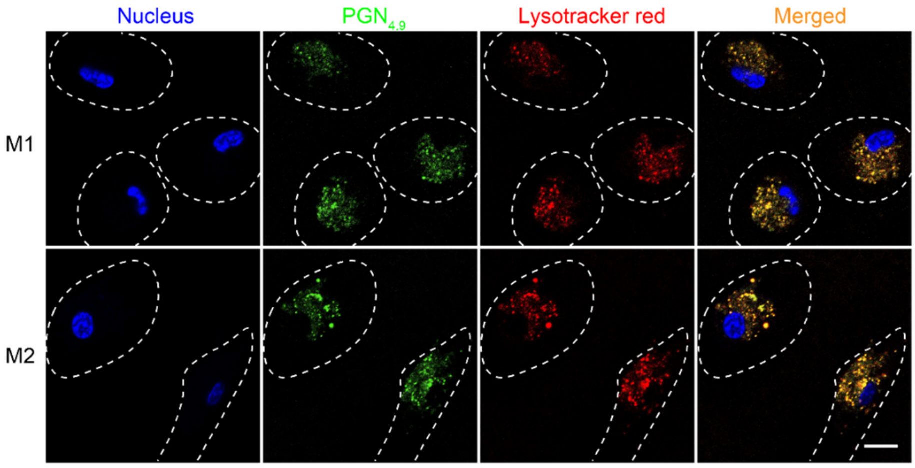

Supplementary Fig. 7. Intracellular distribution of $PGN_{4.9}$ on BMDMs after incubation for 2 h (*n* = 3 experiments)**.** Scale bar, 10 µm.

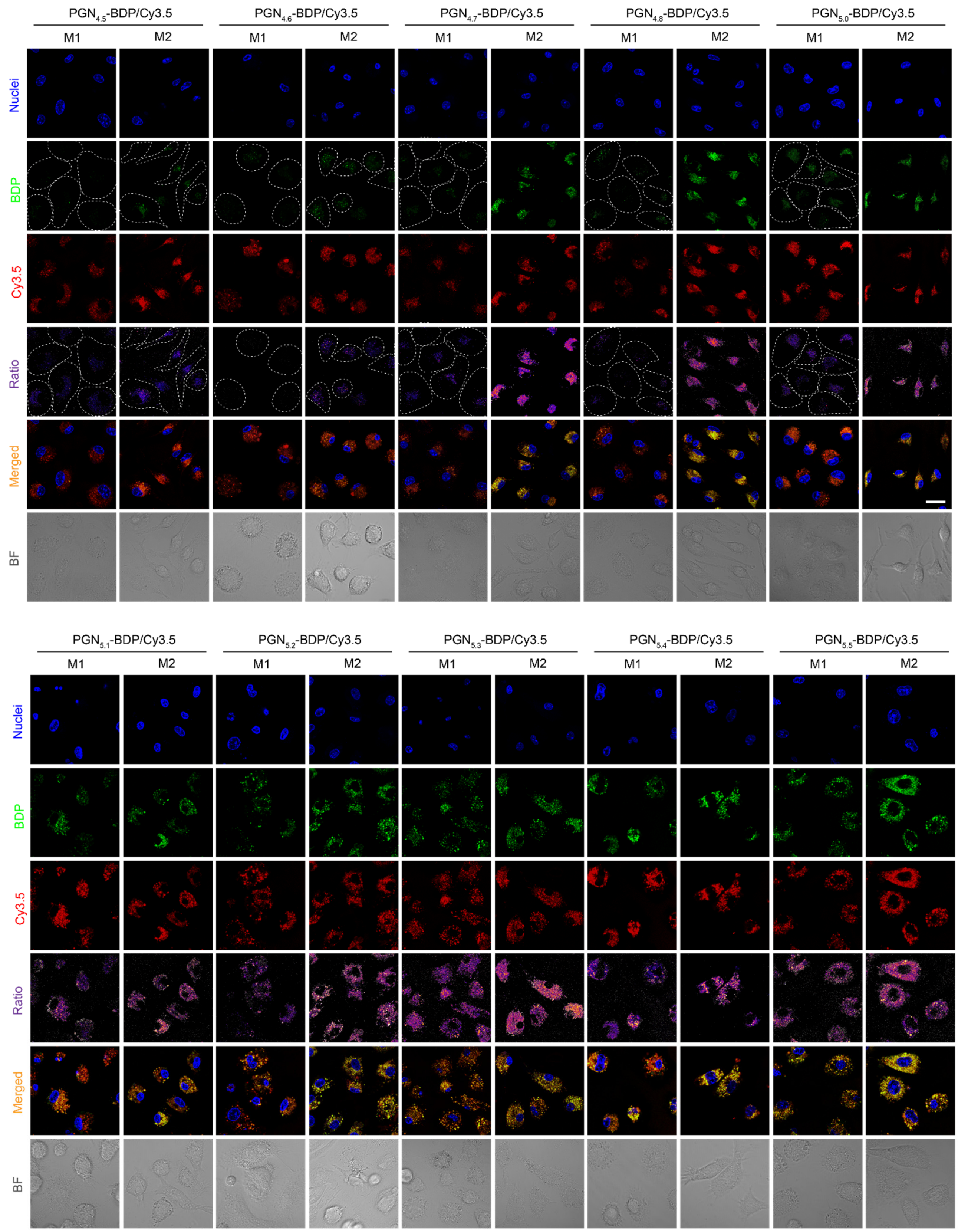

Supplementary Fig. 8. CLSM images of BMDMs treated with PGN library using a pulse-chase assay. M1- and M2-like BMDMs were pulsed with PGN-BDP/Cy3.5 (100 μg mL^{-1}) on the ice for 10 min and then chased at 37 °C for 4 h to colocalize with lysosomes (n = 3 experiments). Green, BDP; red, Cy3.5; and ratiometric images were processed by ImageJ software. Scale bar, 25 μm.

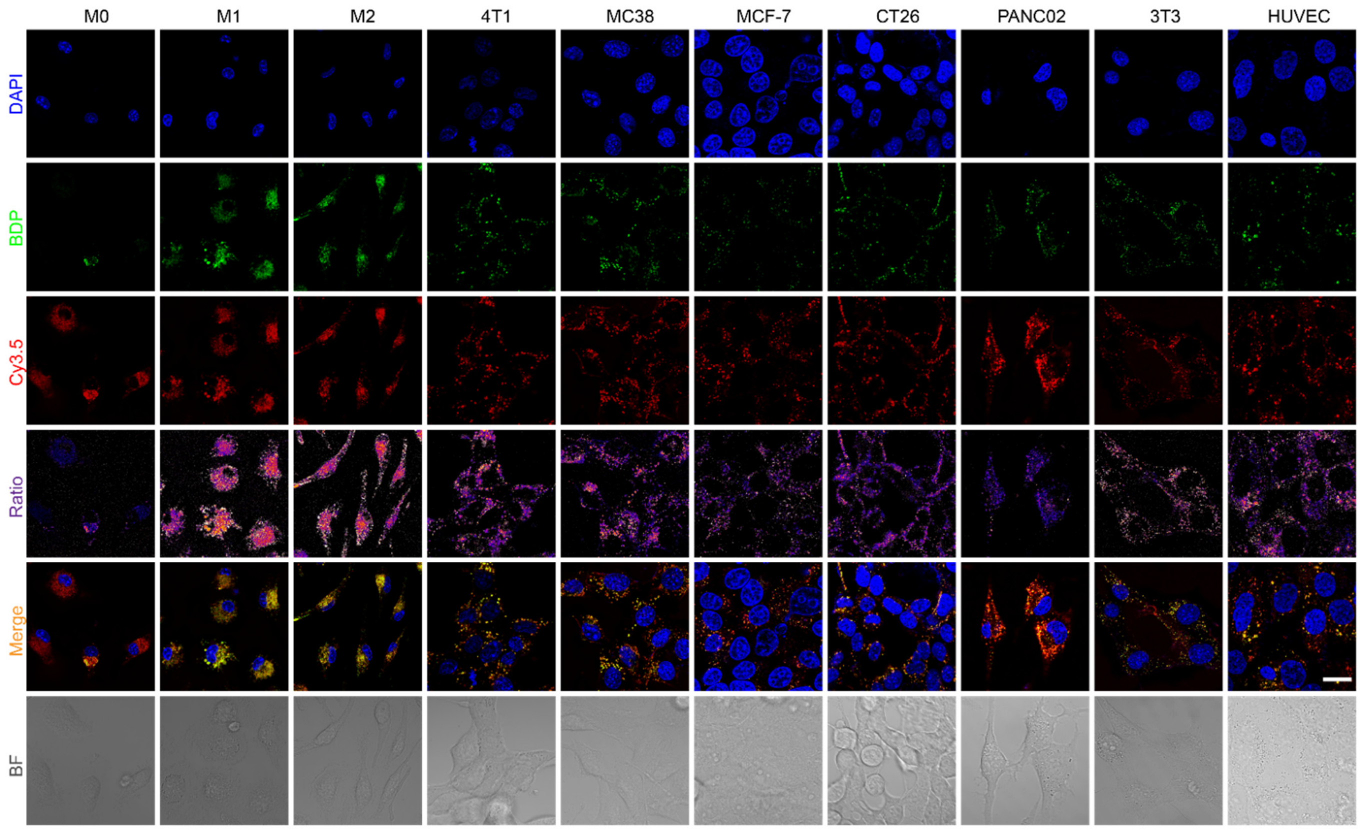

Supplementary Fig. 9. Representative confocal images of different cell lines incubated with $PGN_{6.3}$ via a pulse-chase assay. Cells were pulsed with $PGN_{6.3}$-BDP/Cy3.5 (100 µg mL^{-1}) on the ice for 10 min and then chased at 37 °C for 4 h to colocalize with lysosomes (*n* = 3 experiments). Scale bar, 20 µm.

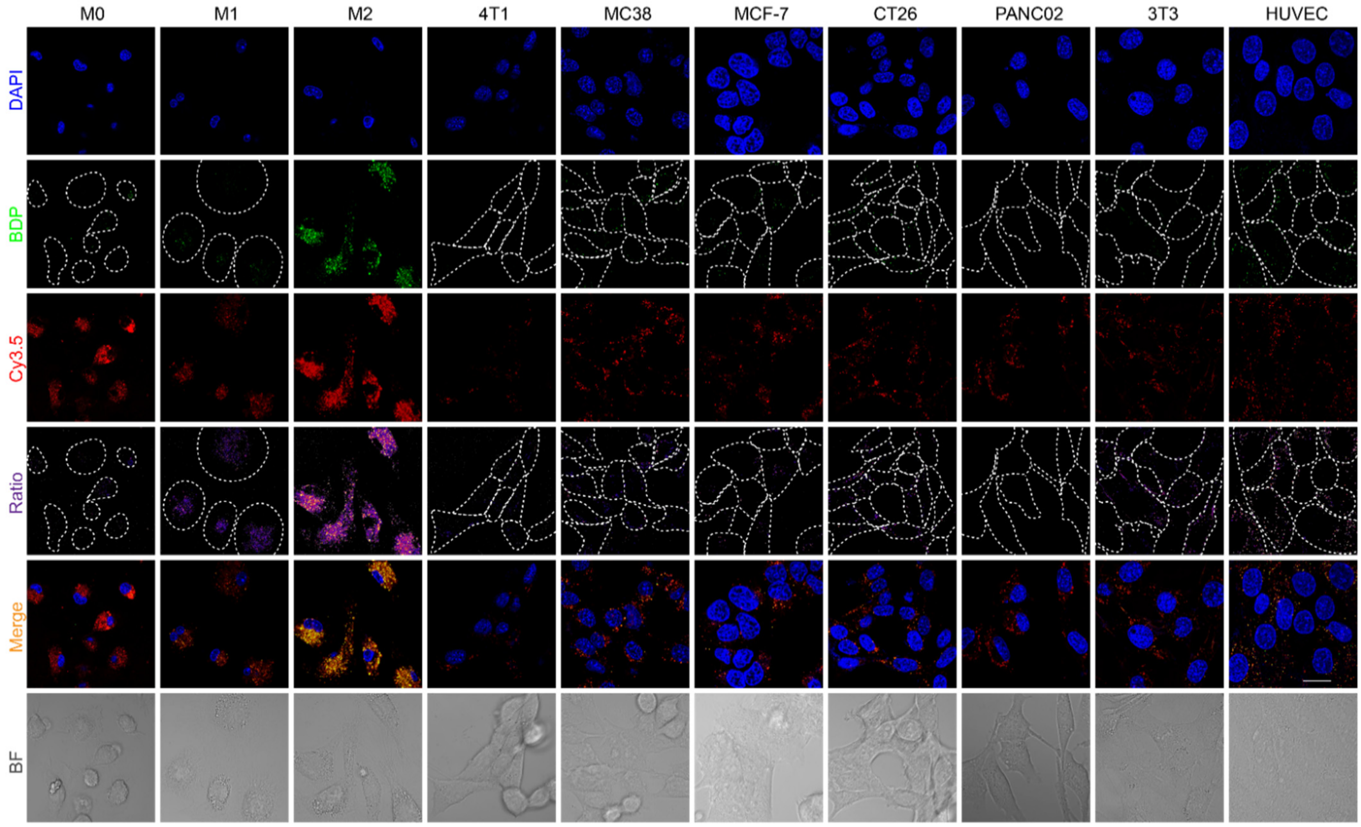

Supplementary Fig. 10. Representative confocal images of different cell lines incubated with $PGN_{4.9}$ via a pulse-chase assay. Cells were pulsed with $PGN_{4.9}$-BDP/Cy3.5 (100 µg mL^{-1}) on the ice for 10 min and then chased at 37 °C for 4 h to colocalize with lysosomes (n = 3 experiments). Scale bar, 20 µm.

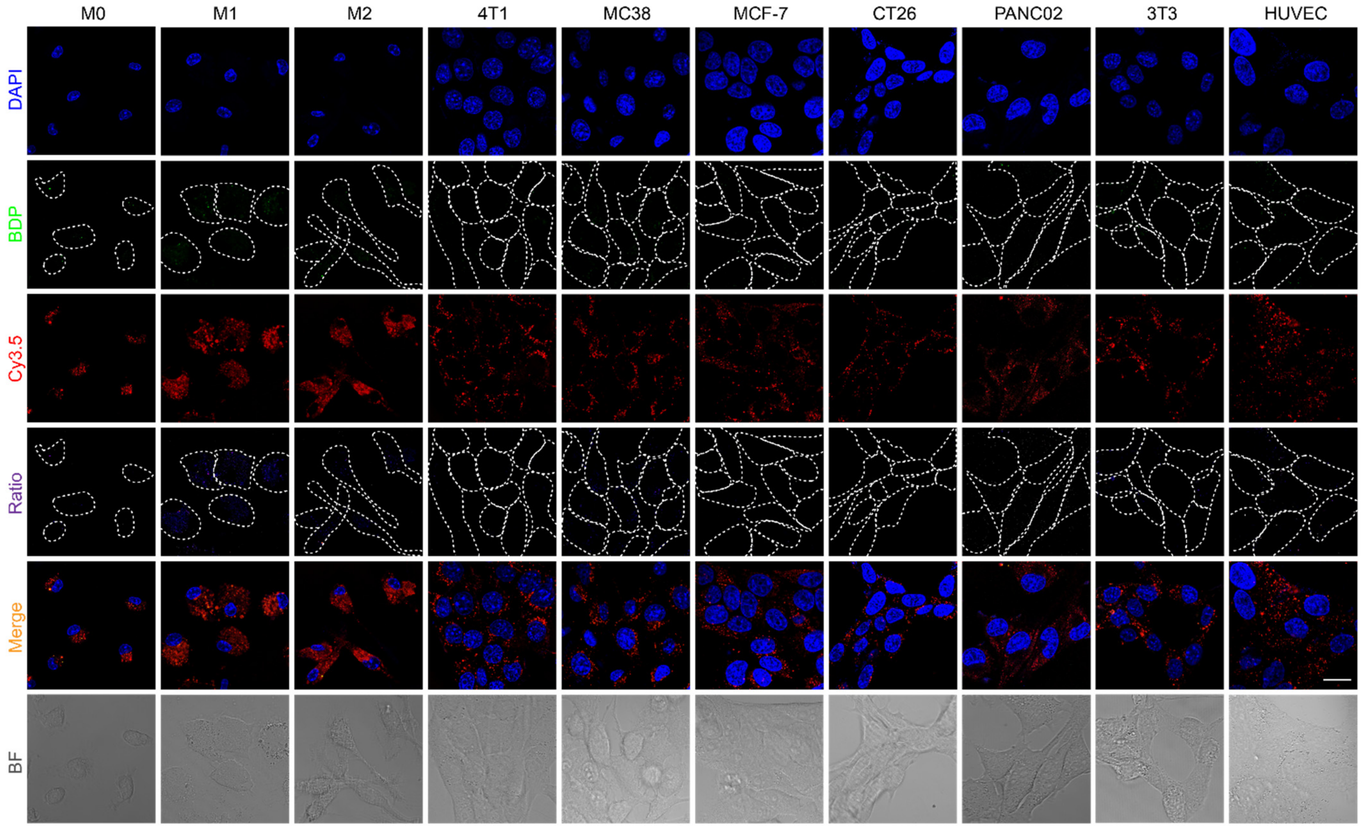

Supplementary Fig. 11. Representative confocal images of different cell lines incubated with $PGN_{4.3}$ via a pulse-chase assay. Cells were pulsed with $PGN_{4.3}$-BDP/Cy3.5 (100 μg mL^{-1}) on the ice for 10 min and then chased at 37 °C for 4 h to colocalize with lysosomes (n = 3 experiments). Scale bar, 20 μm.

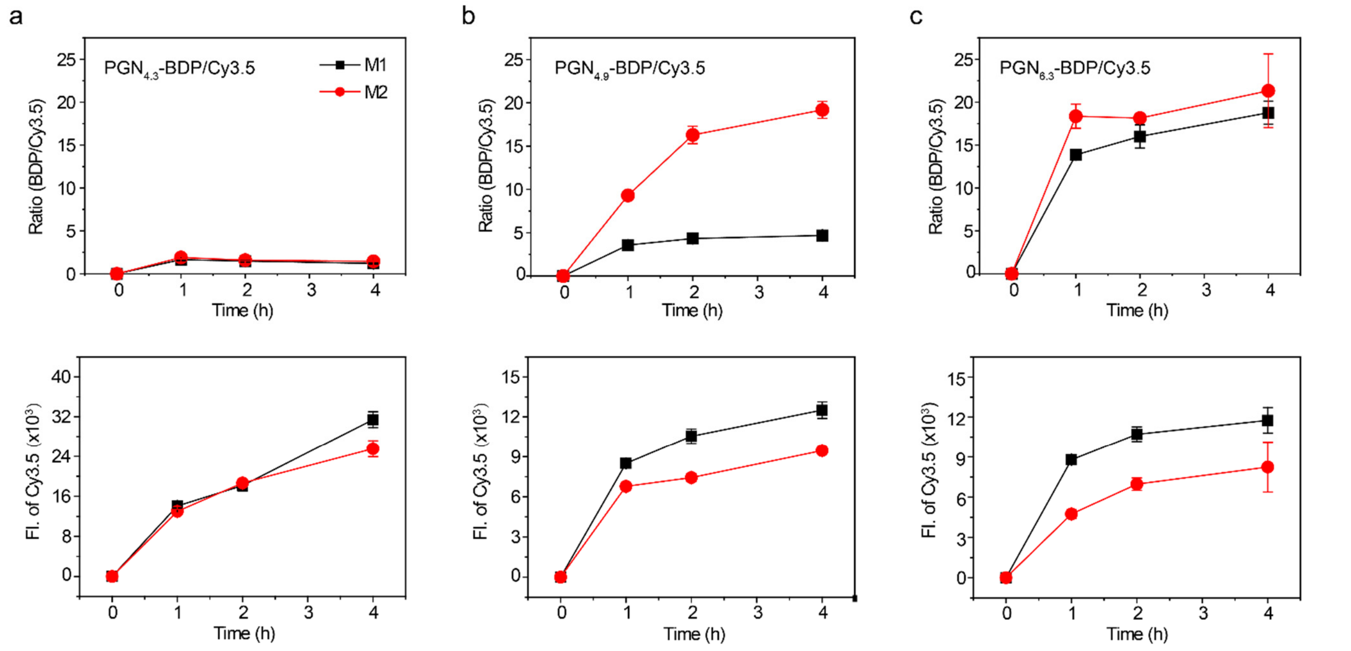

Supplementary Fig. 12. Flow cytometry of BMDMs incubated with $PGN_{4.9}$ in vitro. BMDMs were implanted into 24-well plates and treated with PGN-BDP/Cy3.5 with three different pH_t at 1, 2 and 4 h, respectively. The ratiometric signals represented activation efficacy in response to pH and fluorescence intensity of Cy3.5 represented nanoparticle level of cell uptake. **(a)** $PGN_{4.3}$-BDP/Cy3.5; **(b)** $PGN_{4.9}$-BDP/Cy3.5; **(c)** $PGN_{6.3}$-BDP/Cy3.5 (n = 3 experiments). Source data are provided as a Source Data file.

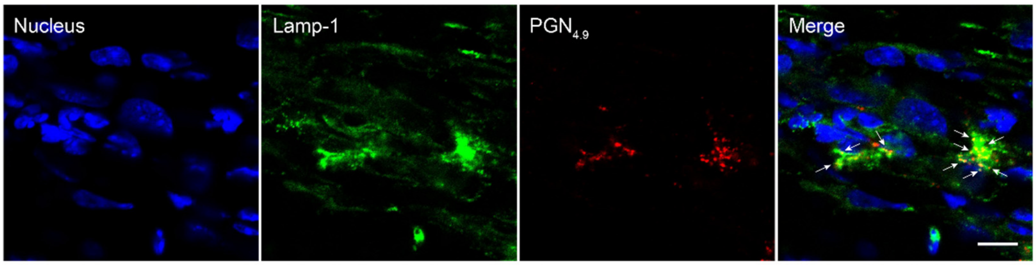

Supplementary Fig. 13. Confocal images of endocytic organelles distribution of PGN$_{4.9}$ nanoprobe in 4T1 tumour-bearing mice. Immunofluorescence staining of Lamp 1 (Green) was performed to label lysosomes (n = 3 experiments). Scale bar, 10 μm.

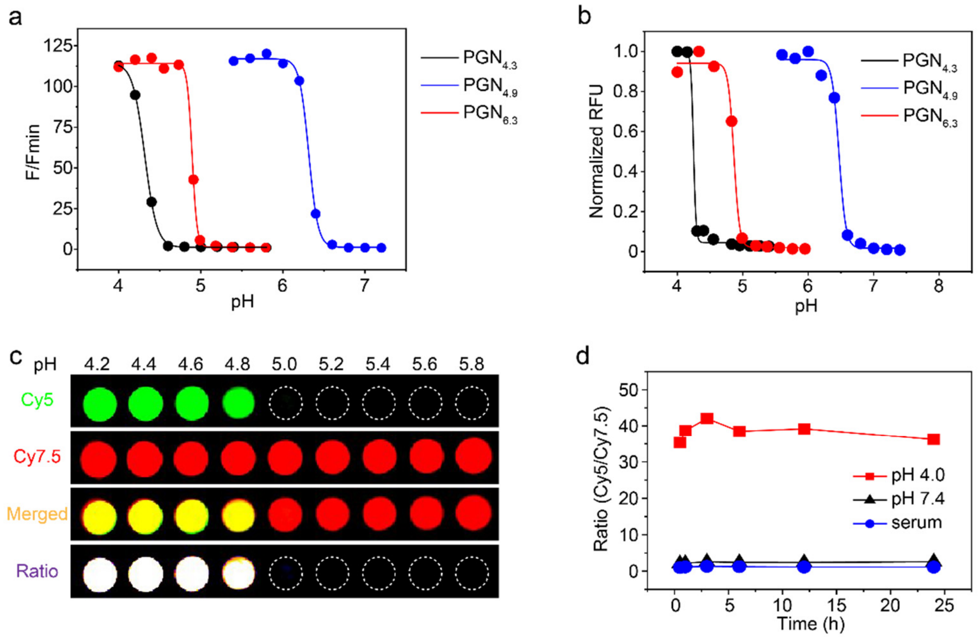

Supplementary Fig. 14. Characterization of PGN nanoparticles labeled with near-infrared fluorophores pairs. (a) Fluorescence activation ratio as the function of pH for PGN-ICG nanoparticles with different pH_t (n = 1 experiment). **(b)** Fluorescence activation ratio as the function of pH for PGN-Cy5/Cy7.5 nanoprobes (n = 1 experiment). **(c)** Fluorescent images of $PGN_{4.9}$-Cy5/Cy7.5 in buffer solutions with different pH in 384-well plate by IVIS Spectrum imaging system ($\lambda_{ex}/\lambda_{em}$: 620 ± 10 nm/ 670 ± 20 nm for Cy5, 780 ± 10 nm/ 845 ± 20 nm for Cy7.5, n = 3 experiments). **(d)** The fluorescence intensity of $PGN_{4.9}$-Cy5/Cy7.5 nanoparticles (100 μg mL^{-1}) in different media at specific time-points (n = 1 experiment).

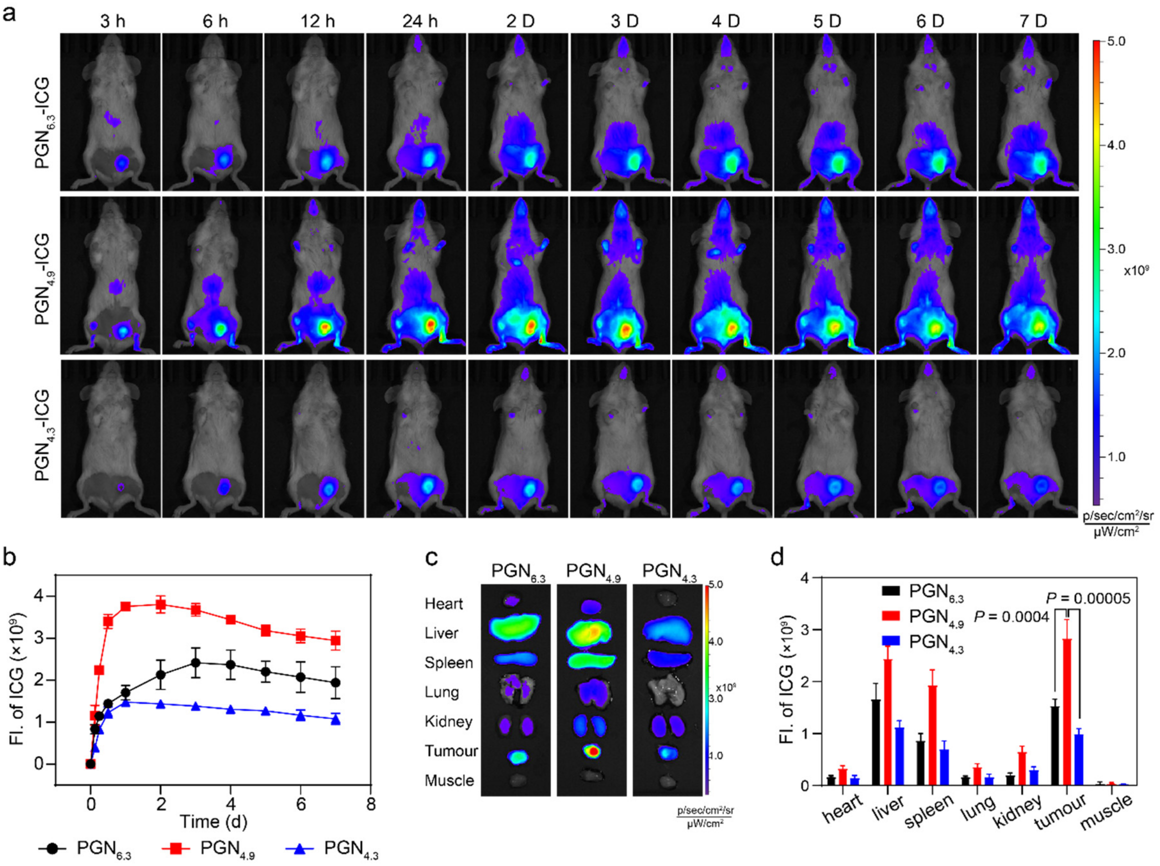

Supplementary Fig. 15. In vivo long-term monitoring of PGNs distribution in orthotopic 4T1 breast tumour xenografts. (a) Representative fluorescent images of 4T1 tumour-bearing mice at selected time-points after intravenous injection of three ICG-conjugated PGNs (20 mg kg^{-1}) by IVIS Spectrum imaging system. **(b)** Time-dependent fluorescence signals were quantified after injection of PGNs. **(c)** Nanoparticle level in excised tumour and major organs at 24 hours after administration of ICG-conjugated PGNs (20 mg kg^{-1}). **(d)** Mean fluorescence intensity of ICG signals in excised tumour and different organs (n = 4 mice, one-way ANOVA followed by Tukey's multiple comparisons test). Source data are provided as a Source Data file.

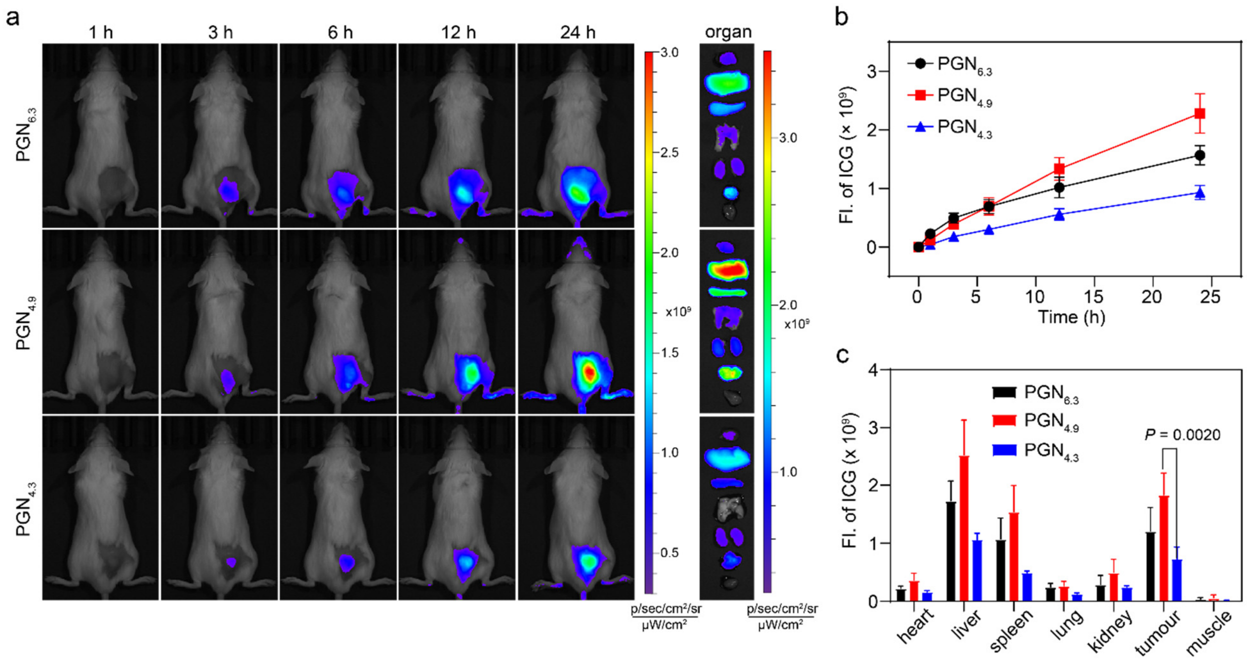

Supplementary Fig. 16. Real-time monitoring of PGNs activation in CT26 colorectal tumour model. (a) BALB/c mice were inoculated subcutaneously with CT26 tumour cells and injected with PGNs-ICG (20 mg kg^{-1}) intravenously. Representative fluorescent images of ICG channel at predesignated time points and ex vivo major organs imaging at 24 h were visualized. **(b)** ICG fluorescence intensity as a function of time in vivo. **(c)** The quantitation of fluorescent signals in dissected organs (n = 4 mice, one-way ANOVA followed by Tukey's multiple comparisons test)). Source data are provided as a Source Data file.

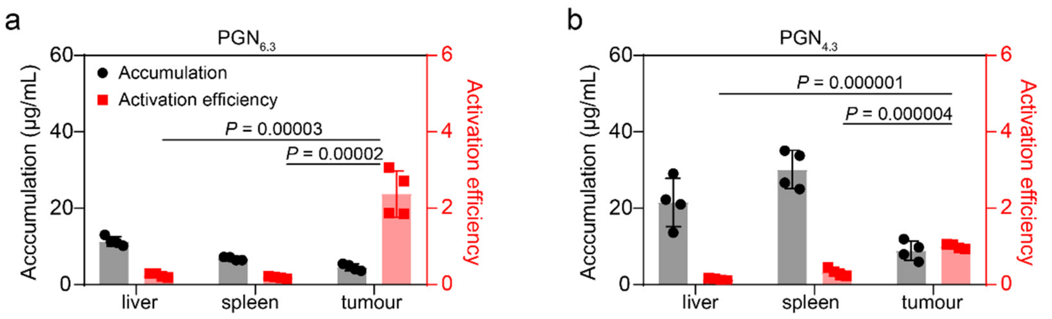

Supplementary Fig. 17. Accumulation level and activation efficiency of **(a)** $PGN_{6.3}$ and **(b)** $PGN_{4.3}$ in dissected livers, spleens and tumours (*n* = 4 mice, one-way ANOVA followed by Tukey's multiple comparisons test). Activation efficiency in different groups was obtained by normalizing to that of $PGN_{4.3}$ in tumour tissues. Source data are provided as a Source Data file.

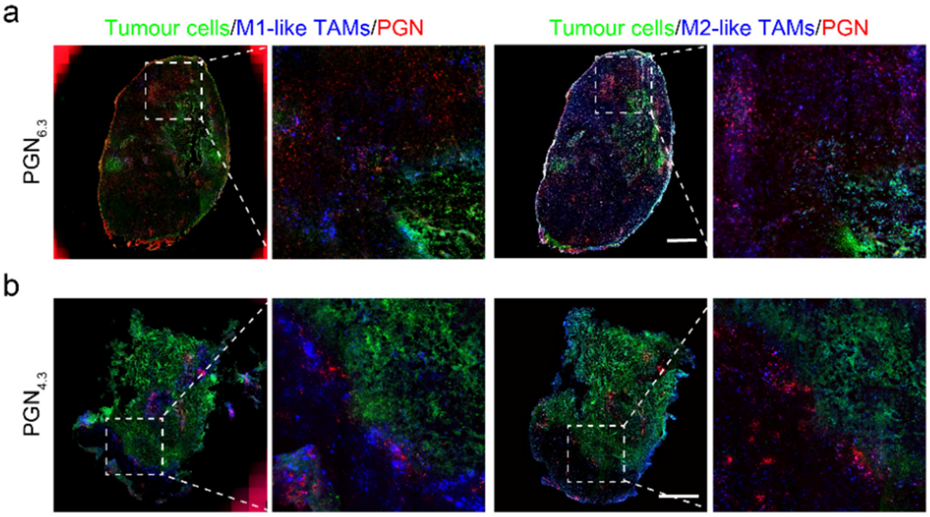

Supplementary Fig. 18. Microscopic distribution of PGNs. Whole-mount tumour adjacent slices of different Cy5-conjugated PGNs distribution from 4T1-GFP tumour-bearing mice at 24 h post-administration (*n* = 3 experiments). **(a)** $PGN_{6.3}$; **(b)** $PGN_{4.3}$. Scale bar, 500 μm.

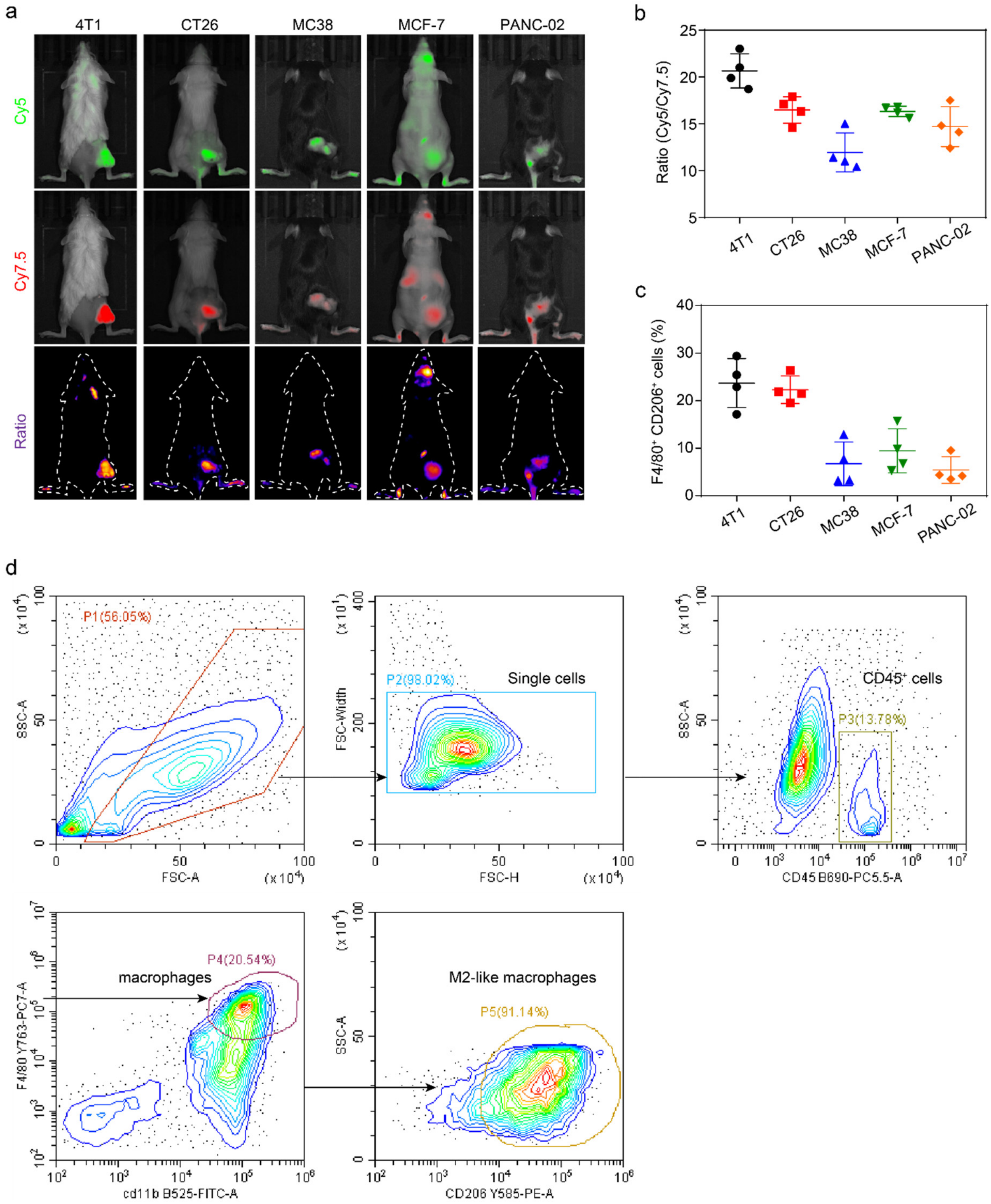

Supplementary Fig. 19. Ratiometric signals as a function of M2-like macrophage content in different tumour models. (a) Representative fluorescent images of mice with five tumour models (4T1, MCF-7, CT26, MC38, PANC-02) were captured at 24 h post-injection of PGN4.9-Cy5/Cy7.5 nanoparticles (20 mg kg^{-1}). **(b)** Ratio values were quantified by fluorescent activation of nanoparticles in vitro using IVIS Spectrum imaging system. **(c)** Percentage of M2-like tumour-associated macrophages in mice were measured by flow cytometry (*n* = 4 mice). **(d)** FACS gating strategy for stratification of M2-like macrophages in different tumour models.

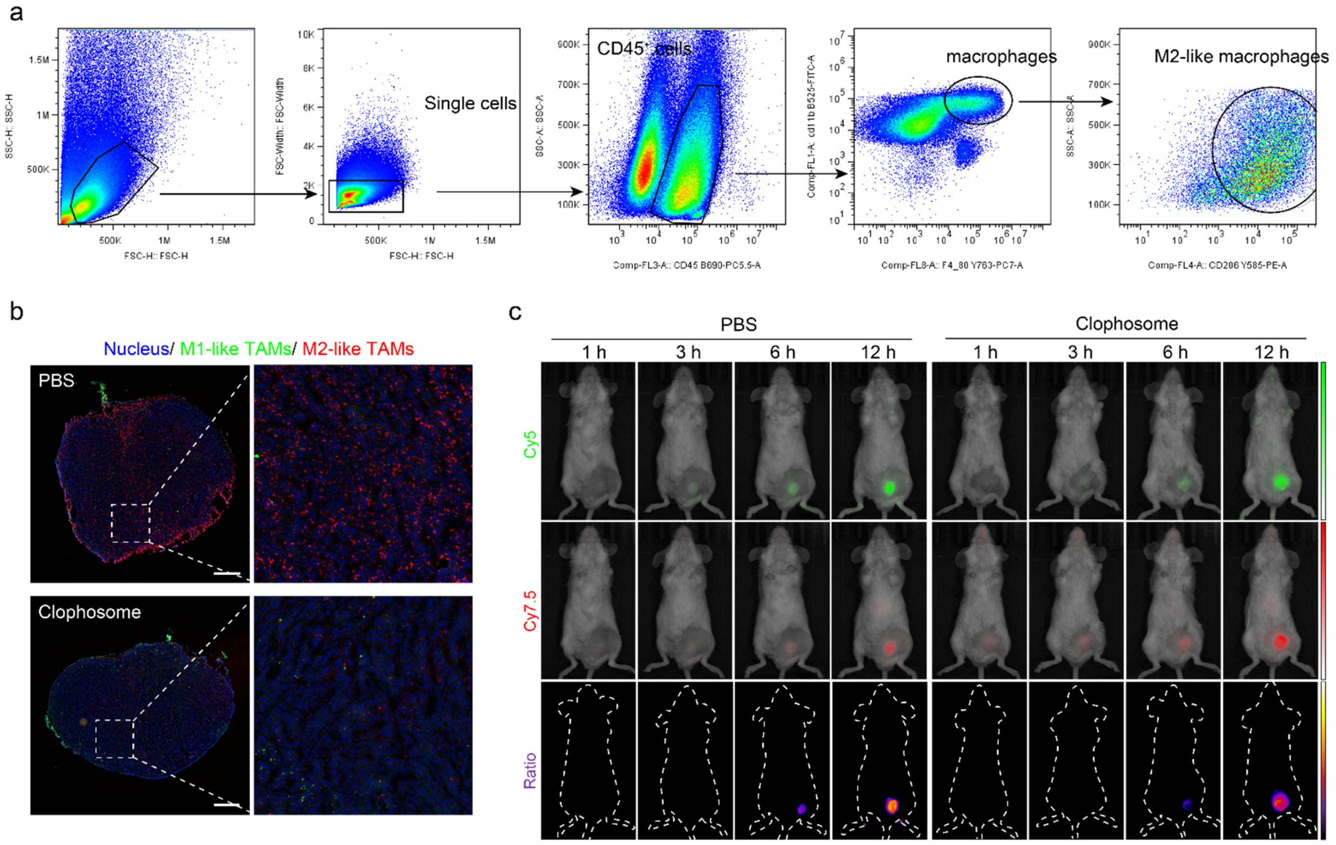

Supplementary Fig. 20. The effect of macrophage depletion on PGN$_{4.9}$ activation in 4T1 tumour-bearing mice. (a) FACS gating strategy for stratification of M2-like TAMs in mice with or without treatment of clophosome. **(b)** Immunofluorescence staining of iNOS (green), CD206 (red) and Nucleus (blue) of tumour tissue with intravenous injection of PBS or clophosome (*n* = 3 experiments). Scale bar, 800 µm. **(c)** Time-dependent fluorescent images of 4T1 tumour-bearing mice pretreated with PBS or Clophosome after intravenous injection of PGN$_{4.9}$-Cy5/Cy7.5 (20 mg kg^{-1}). Ratiometric images were visualized by via ImageJ software (NIH) (*n* = 4 mice for PBS group; *n* = 3 mice for clophosome group).

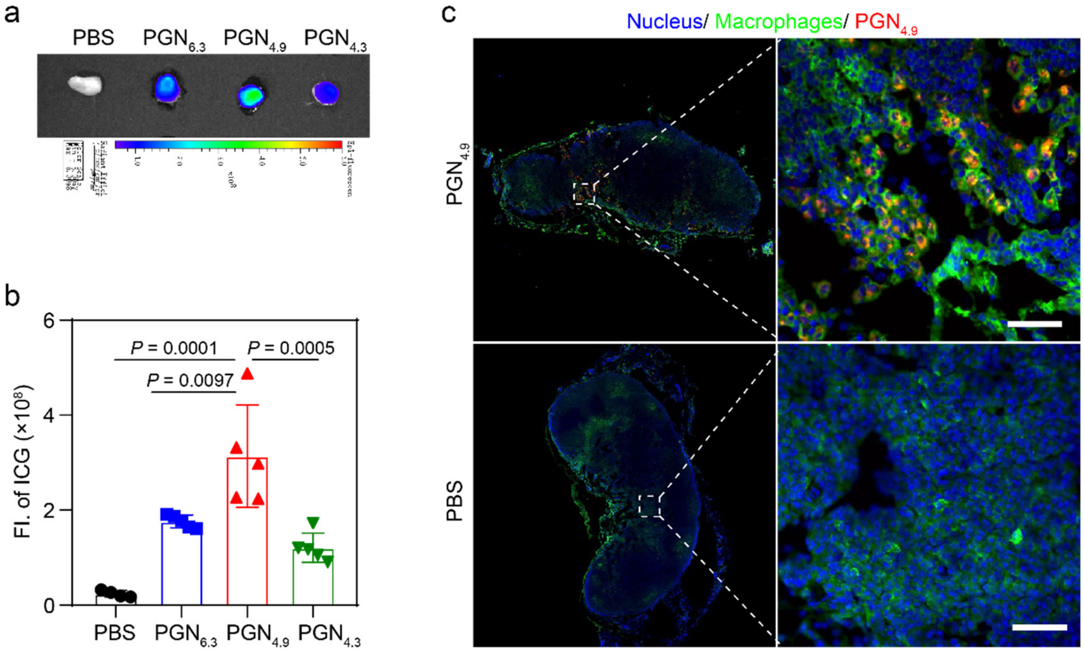

Supplementary Fig. 21. Biodistribution of PGNs in lymph nodes from 4T1 tumour-bearing mice. (a) Nanoparticle level in excised lymph nodes at 24 hours after administration of ICG-conjugated PGNs (20 mg kg^{-1}) (*n* = 3 experiments). **(b)** Mean fluorescence intensity of ICG signals in lymph nodes (*n* = 4 for PBS group; *n* = 5 for other groups, one-way ANOVA followed by Tukey's multiple comparisons test). **(c)** Whole-mount images of lymph node slices at 24 h post-administration of Cy5-labelled $PGN_{4.9}$ and control group. Scale bar, 50 μm. The macrophages were stained with anti-F4/80 antibody (*n* = 3 experiments). Source data are provided as a Source Data file.

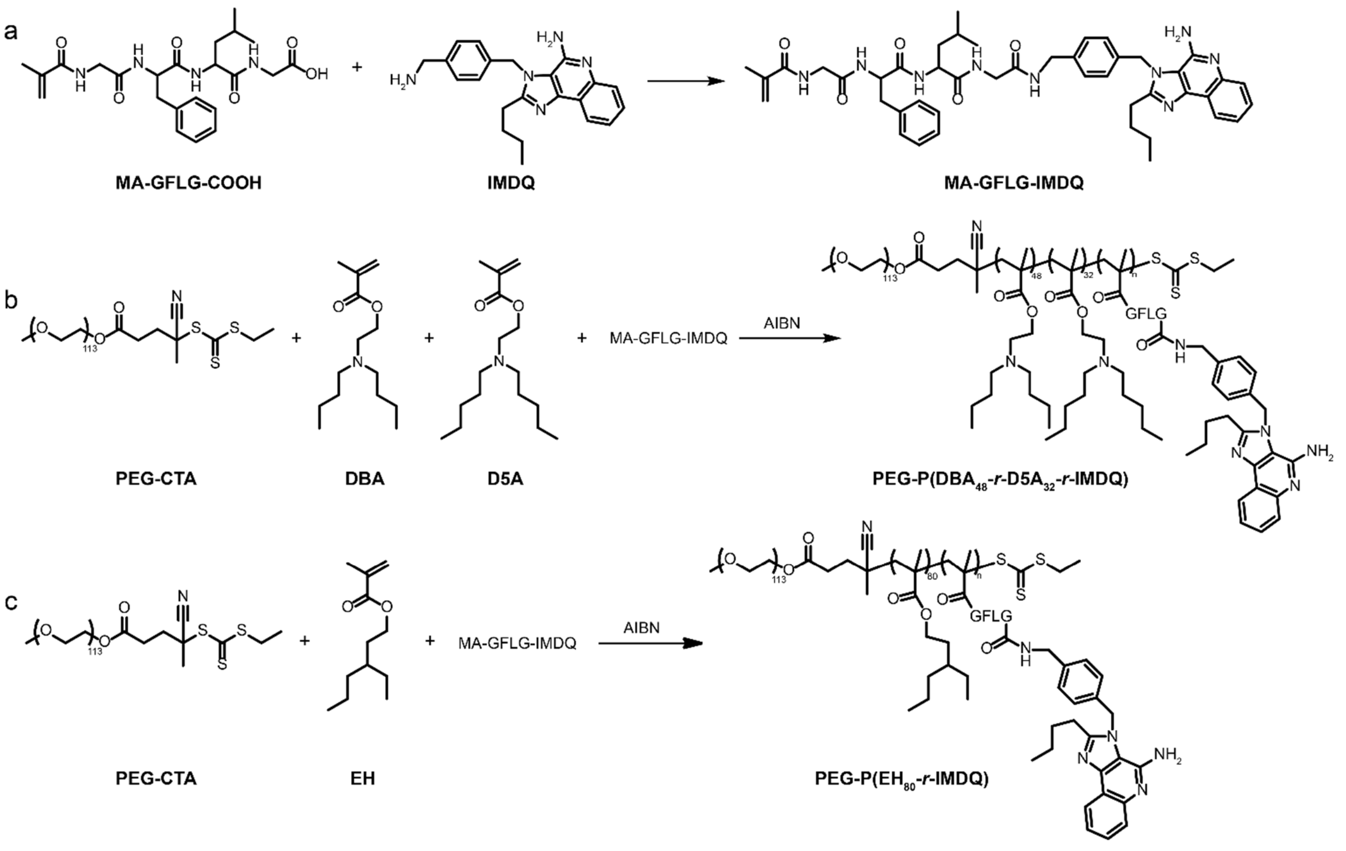

Supplementary Fig. 22. Synthetic routes of AND-gate drug-conjugated copolymers. (a) The synthesis of cathepsin B-sensitive monomer MA-GFLG-IMDQ. **(b)** PEG-*b*-P(DBA_{48}-*r*-$D5A_{32}$-*r*-IMDQ) and **(c)** PEG-*b*-P(EH_{80}-*r*-IMDQ) copolymers were synthesized by RAFT polymerization.

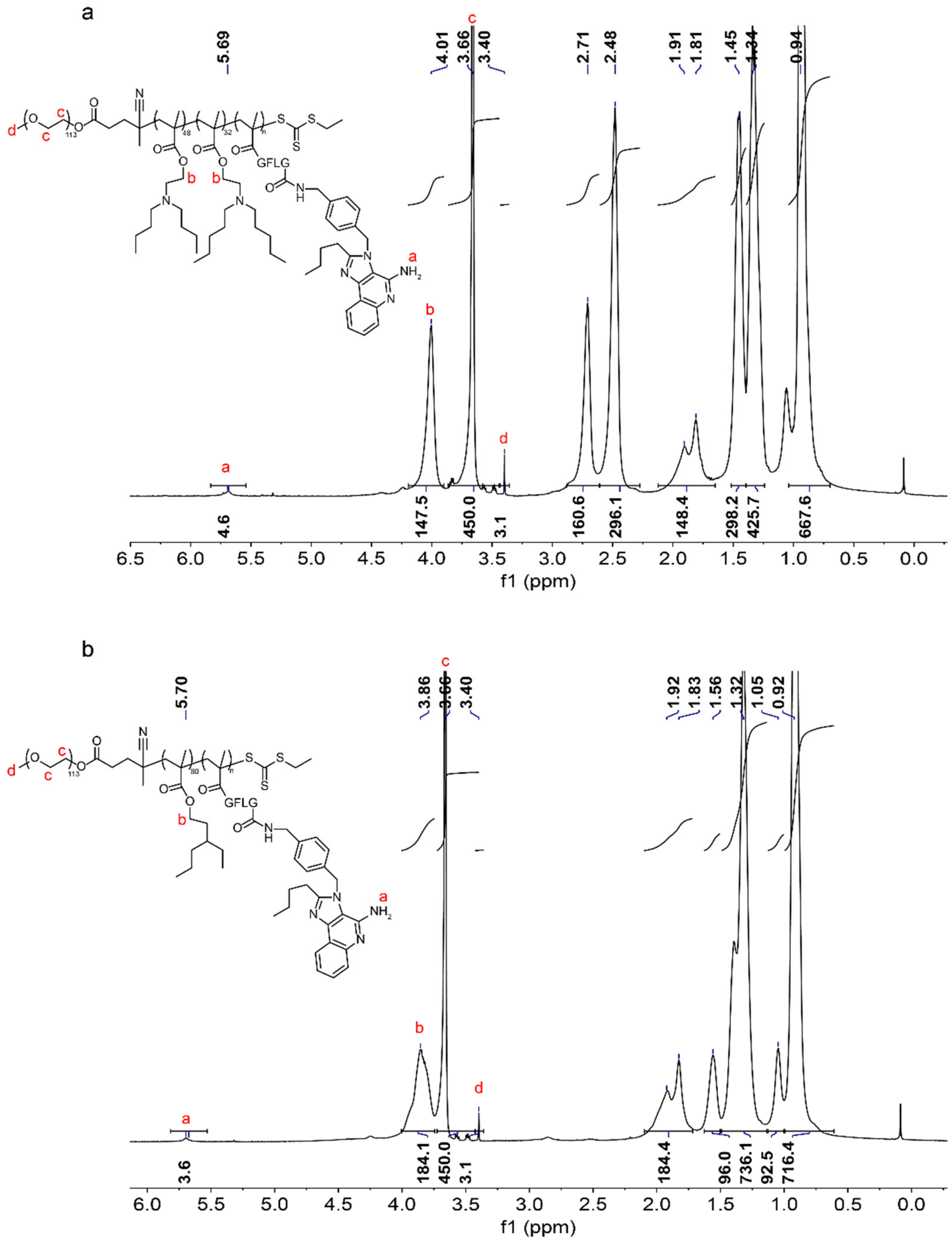

Supplementary Fig. 23. ^{1}H-NMR spectra of **(a)** PEG-*b*-P(DBA$_{48}$-D5A$_{32}$-*r*-GFLG-IMDQ). ^{1}H NMR (400 MHz, CDCI3): δ 5.69 (s, 4.60H), 4.00 (m, 147H), 3.66 (m, 450H), 3.40 (s, 3H), 2.71 (m, 148H), 2.48 (m, 296H), 2.10-1.66 (m, 148H), 1.45 (m, 298H), 1.34 (m, 425H), 0.94 (m, 667H). **(b)** PEG-*b*-P(EH$_{80}$-*r*-GFLG-IMDQ). ^{1}H NMR (400 MHz, CDCI3): δ 5.70 (s, 3.56H), 3.86 (m, 184H), 3.66 (m, 450H), 3.40 (s, 3H), 2.10-1.72 (m, 184H), 1.56 (m, 96H), 1.49-1.16 (m, 736H), 1.05 (m, 92H), 0.92 (m, 716H).

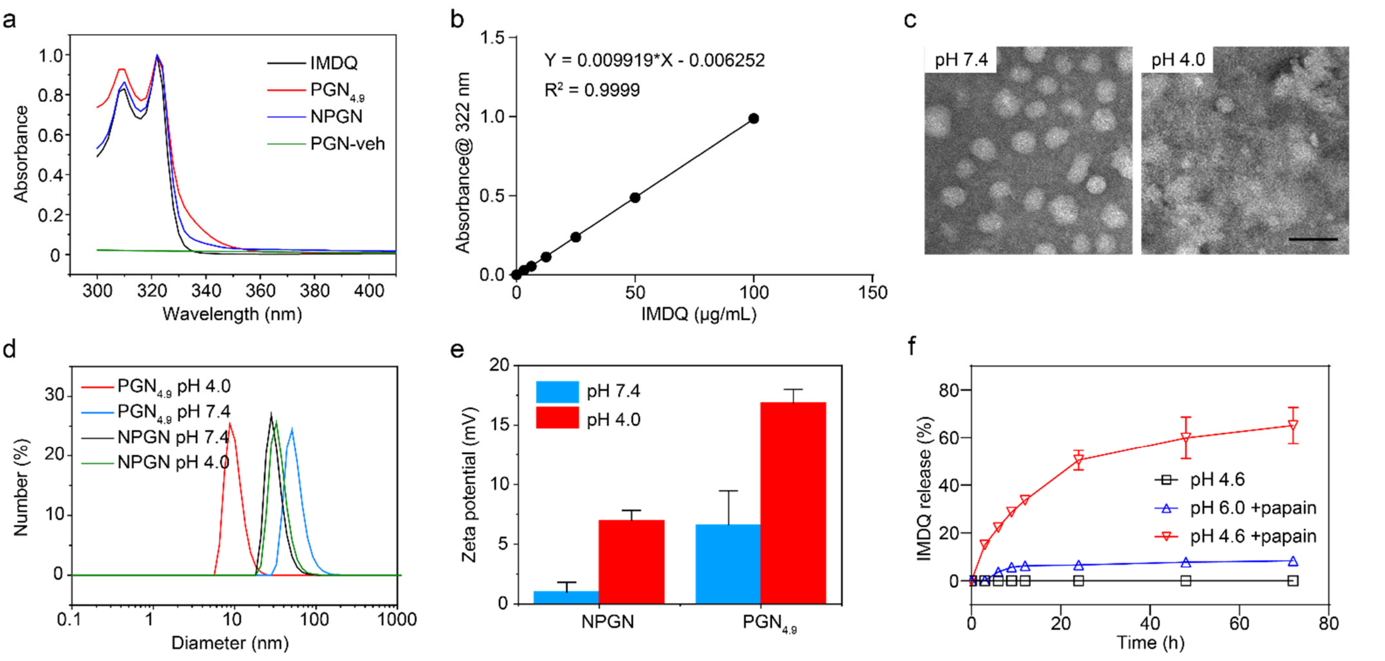

Supplementary Fig. 24. Characterization of $PGN_{4.9}$ and NPGN nanoadjuvants in vitro. (a) UV-Vis spectra of free IMDQ, $PGN_{4.9}$, NPGN nanoadjuvants, and PGN-vehicle in methanol solution (*n* = 1 experiment). **(b)** The standard curve of IMDQ based on UV-Vis absorption at 322 nm. The drug loading efficiency was calculated using the standard curve. **(c)** TEM images, **(d)** Particle size distribution and **(e)** zeta potentials of $PGN_{4.9}$ and NPGN nanoadjuvants (800 μg mL^{-1}) at pH 4.0 and 7.4 (*n* = 3 experiments). Scale bars, 100 nm. **(f)** In vitro IMDQ release profile of $PGN_{4.9}$ nanoadjuvant in different conditions (*n* = 3 experiments). Source data are provided as a Source Data file.

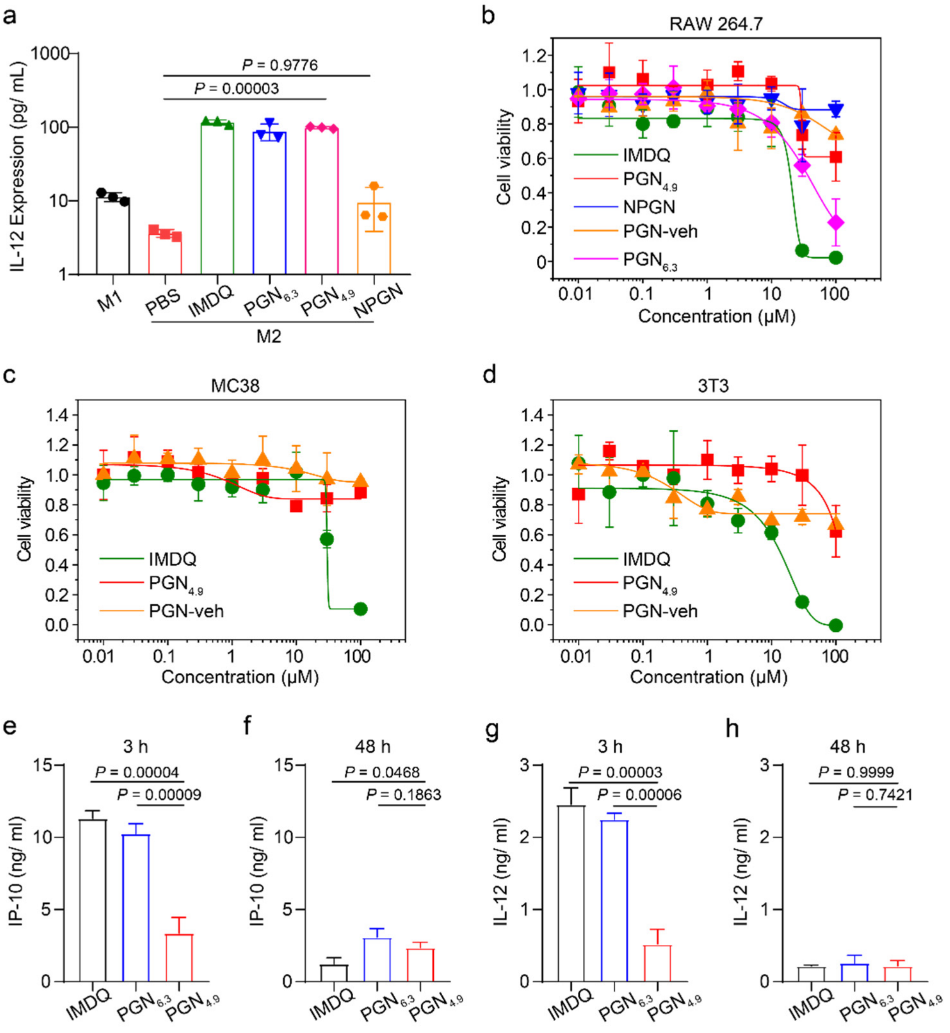

Supplementary Fig. 25. Proinflammatory cytokines and acute systemic toxicity analysis. (a) ELISA assay of IL-12 secreted from BMDMs pretreated with IMDQ preparations (equivalent to 10 µM IMDQ) for 24 hours (n = 3 experiments). Cell viability assay of free IMDQ, PGN4.9 and PGN4.9 in **(b)** RAW264.7 macrophages, **(c)** MC38 tumour cells, and **(d)** 3T3 fibroblast cells. Cells were treated with various preparations at a series of concentrations in incomplete DMEM medium for 24 h followed by MTT assay (n = 6 experiments). **(e, f)** A typical proinflammatory cytokine IP-10 was measured from serum of C57BL/6 mice injected intravenously with free IMDQ, PGN6.3 and PGN4.9 for 3 h and 48 h (equivalent to 2 mg kg^{-1} IMDQ). **(g, h)** The content of IL-12 was determined by ELISA kits for 3 h and 48 h as well (n = 3 mice, one-way ANOVA followed by Tukey's multiple comparisons test). Source data are provided as a Source Data file.

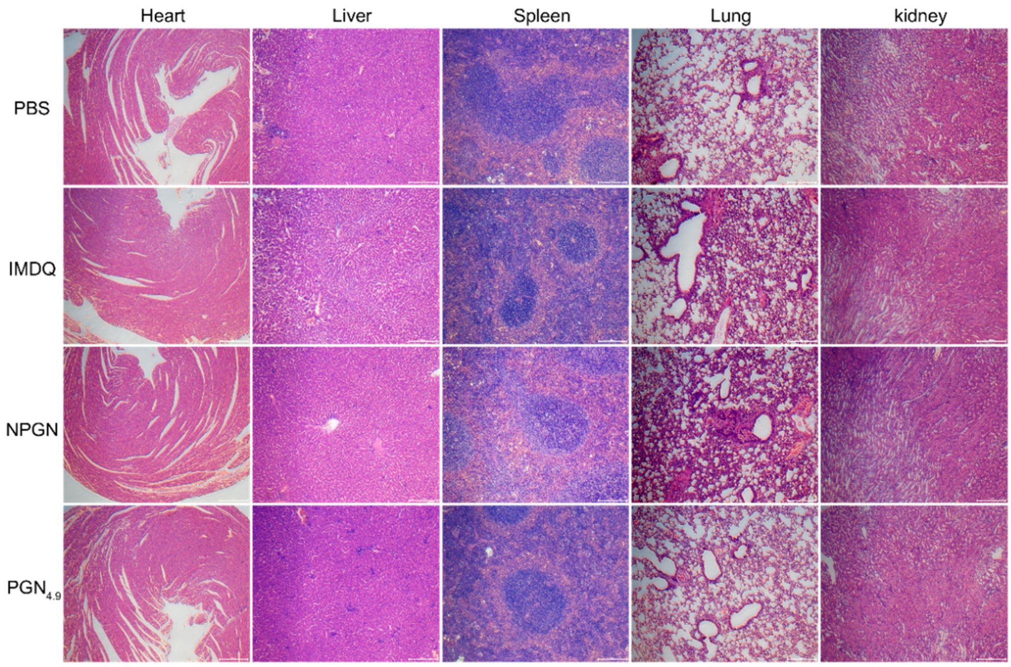

Supplementary Fig. 26. Representative histological features of hearts, livers, spleens, lungs, and kidneys of mice with different treatments 10 days after the last treatment (*n* = 3 experiments). Scale bar, 200 μm.

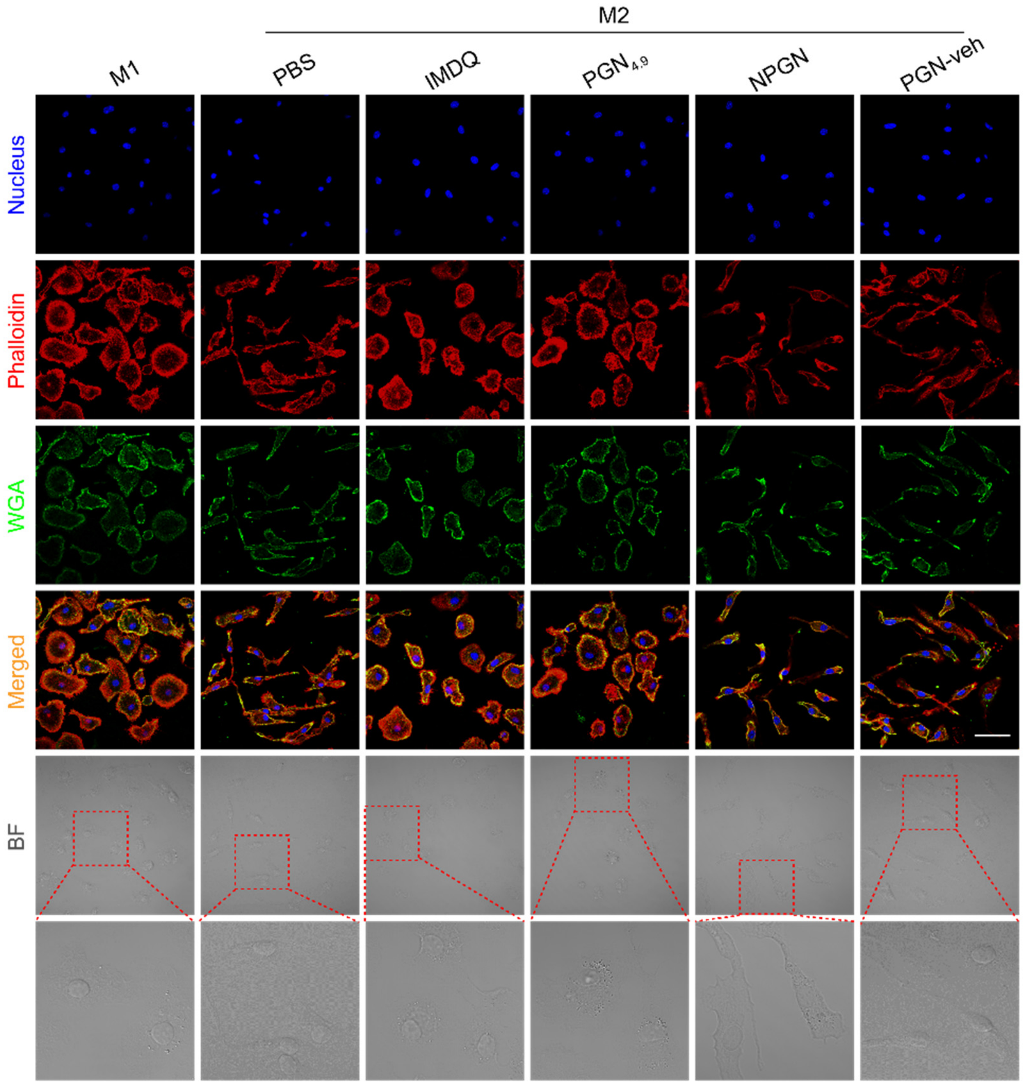

Supplementary Fig. 27. Changes of morphological phenotype in BMDMs. Representative CLSM of BMDMs pretreated with or without IMDQ formulations, followed by staining with WGA and Phalloidin for cell membrane and cytoskeleton, respectively (*n* = 3 experiments). Green, FITC-WGA; red, TRITC-Phalloidin; blue, Hoechst 33342; Grey, bright field. Scale bars, 50 µm.

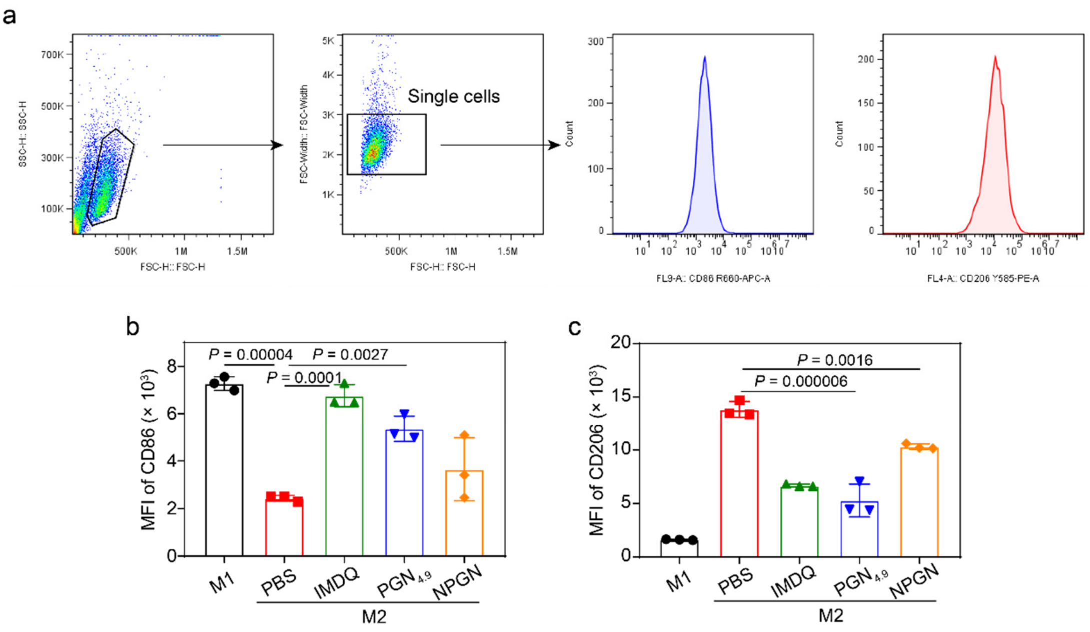

Supplementary Fig. 28. Repolarization of M2-like macrophage to M1-like phenotype by PGN$_{4.9}$ in vitro. (a) FACS gating strategy for the expression of CD86 and CD206 in BMDMs. Flow cytometry results of the expression of M1-related marker CD86 **(b)** and M2-related marker CD206 **(c)** in M2-like BMDMs derived from C57BL/6 mice (*n* = 3 experiments, one-way ANOVA followed by Tukey's multiple comparisons test). Source data are provided as a Source Data file.

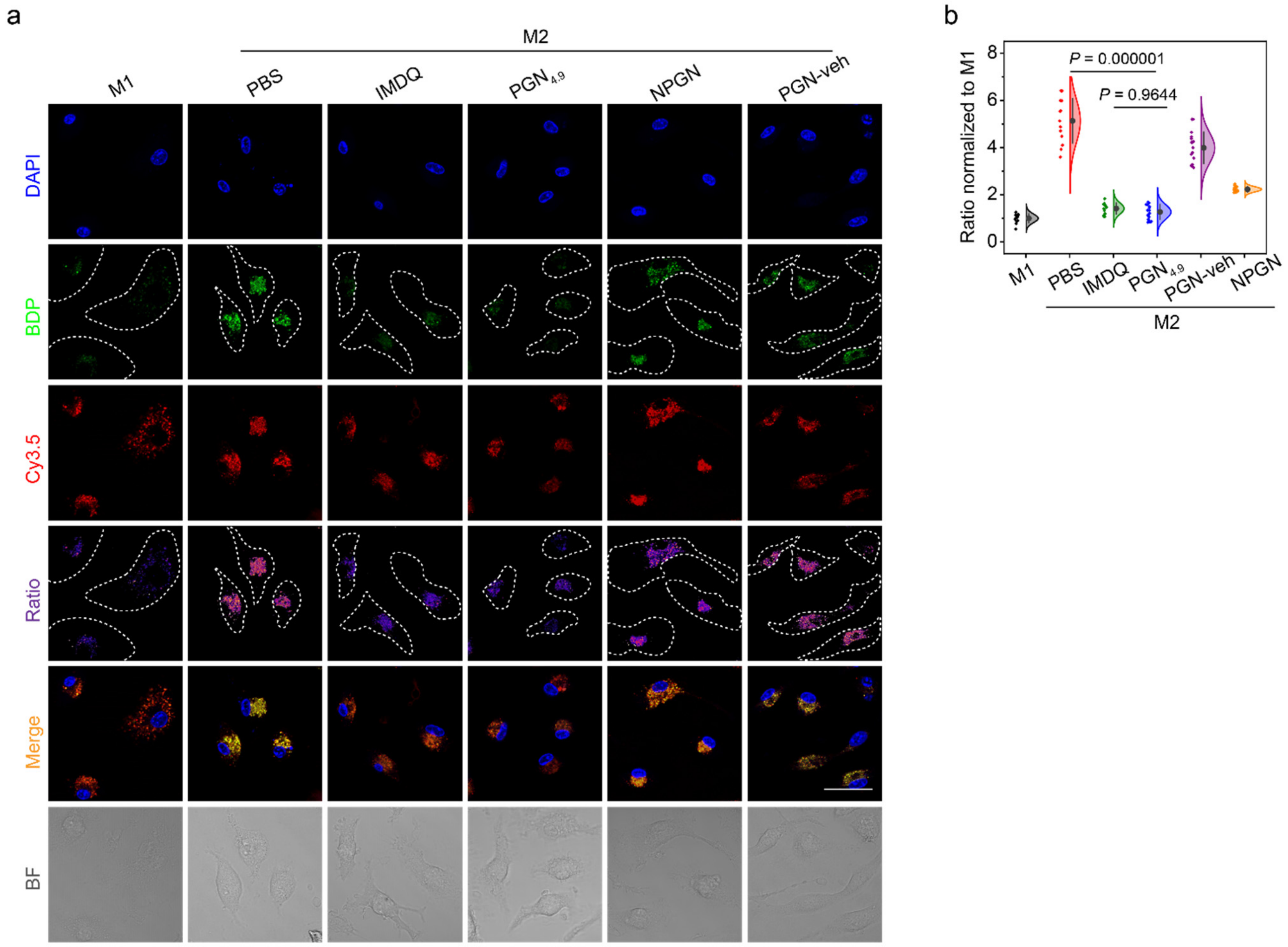

Supplementary Fig. 29. Confocal images of pretreated BMDMs incubated with PGN4.9 nanoreporters. **(a)** M2-like BMDMs were pretreated with various IMDQ groups for 24 h at 37 °C. BMDMs were then pulsed with PGN4.9-BDP/Cy3.5 nanoparticles (100 μg mL^{-1}) on the ice for 10 min and then chased at 37 °C for 4 h to colocalize with lysosomes (*n* = 3 experiments). Scale bar, 20 μm. **(b)** The ratiometric signals of M2-like BMDMs from panel **a** normalized to that of M1-like BMDMs (*n* = 13 cells for M2 and IMDQ groups; *n* = 15 cells for M1 group; *n* = 21 cells for NPGN group; *n* = 17 cells for other groups, one-way ANOVA followed by Tukey's multiple comparisons test). Source data are provided as a Source Data file.

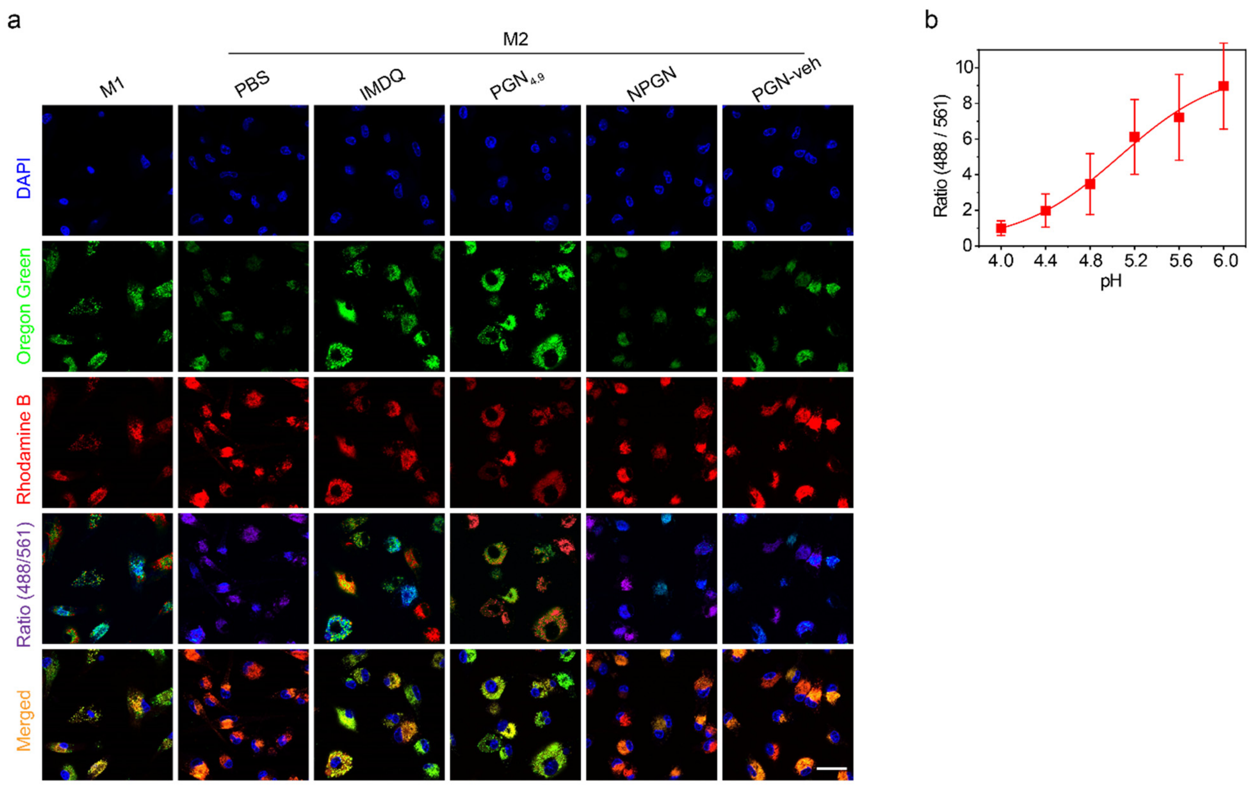

Supplementary Fig. 30. The changes of lysosomal pH in M2-like BMDMs treated with IMDQ formulations. (a) Representative confocal images of M2-like BMDMs incubated with different IMDQ nanoparticles for 24 h, followed by the fluorescent dextran pulse-chase assay (*n* = 3 experiments). Scale bar, 25 μm. **(b)** The fitting standard curve of lysosomal pH value in bone marrow derived macrophages (*n* = 10 cells).

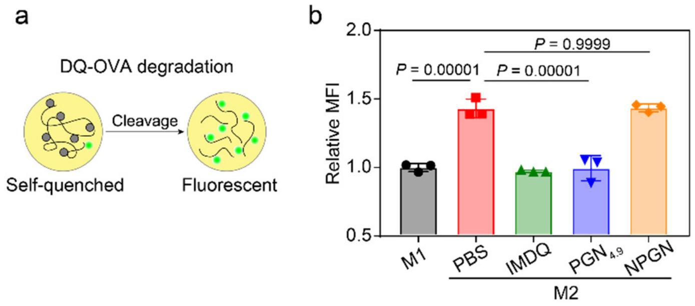

Supplementary Fig. 31. Evaluation of protein degradation assays. (a) Schematic illustration of ovalbumin degradation assay. **(b)** Mean fluorescence intensity of DQ-OVA in BMDMs upon treatment with different IMDQ groups (equivalent to 10 μM IMDQ) for 24 h by flow cytometry (n = 3 experiments, one-way ANOVA followed by Tukey's multiple comparisons test). Source data are provided as a Source Data file.

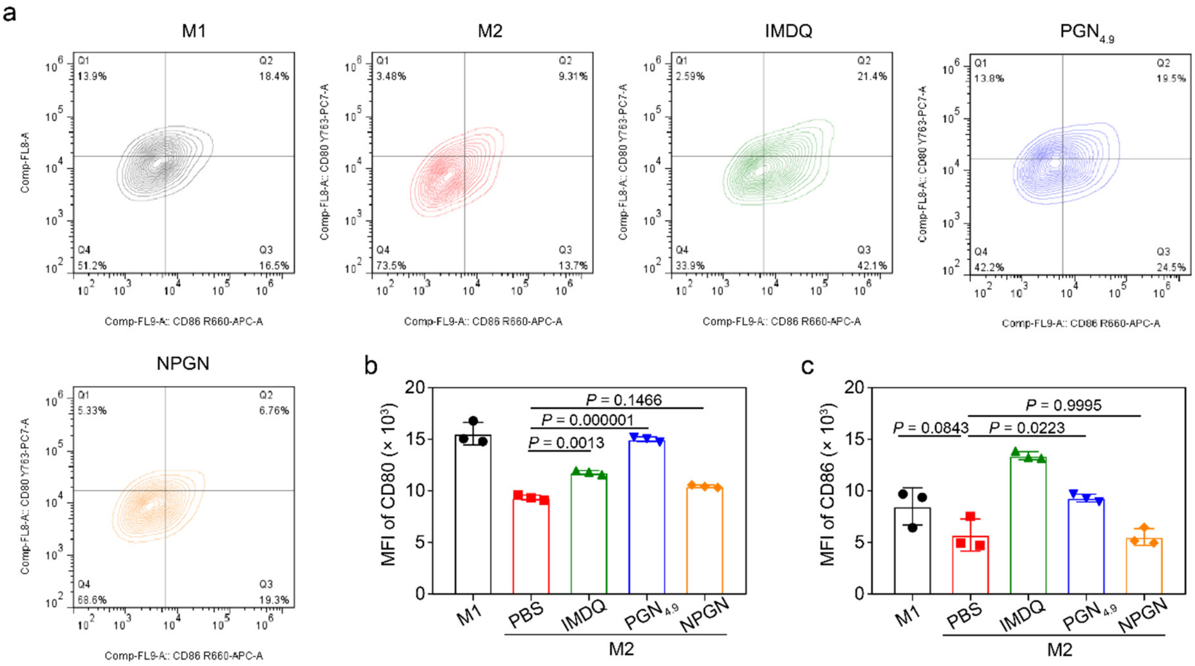

Supplementary Fig. 32. Up-regulation of costimulators in BMDMs for antigen presentation. (a) Representative flow cytometry plots of $CD80^{+}CD86^{+}$ cells from BMDMs treated with different IMDQ formulations (equivalent to 10 μM IMDQ). **(b)** Mean fluorescence intensity of CD80 in different groups. **(c)** Mean fluorescence intensity of CD86 in different groups (n = 3 experiments, one-way ANOVA followed by Tukey's multiple comparisons test). Source data are provided as a Source Data file.

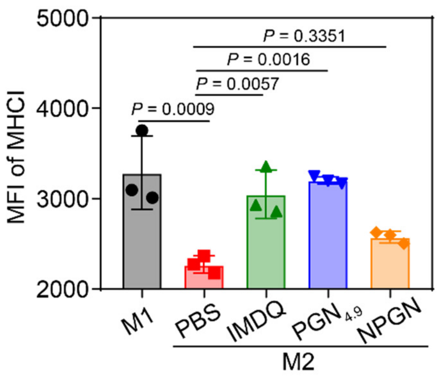

Supplementary Fig. 33. The expression of MHC-I molecules on BMDMs. Mean fluorescence intensity of H-2K^d MHC class I molecules expressed on cell membranes after treatment with different IMDQ formulations (equivalent to 10 μM IMDQ) for 24 h (n = 3 experiments, one-way ANOVA followed by Tukey's multiple comparisons test). Source data are provided as a Source Data file.

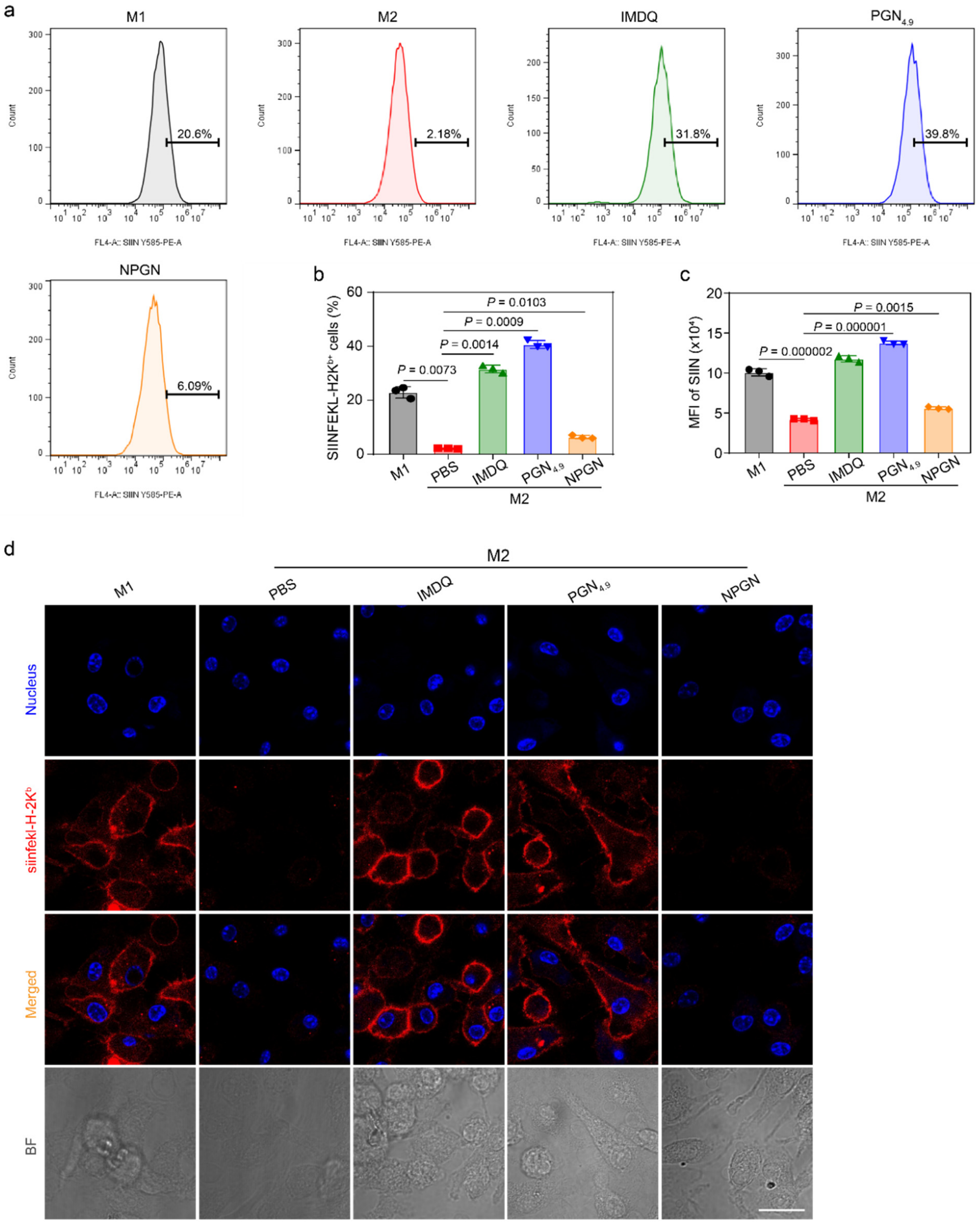

Supplementary Fig. 34. Effect of various IMDQ formulations on antigen cross-presentation. BMDMs treated with different IMDQ groups and SIINFEKL peptide for 24 h. **(a)** Representative flow cytometry plots and **(b)** quantification of $OVA_{257\text{-}264}$-positive BMDMs. **(c)** Expression level of SIINFEKL-H-2K^b-PE on BMDMs (n = 3 experiments). **(d)** Confocal images of SIINFEKL presenting on BMDMs (n = 3 experiments). Scale bar, 25 μm. Statistical significance was analyzed by one-way ANOVA followed by Tukey's multiple comparisons test. Source data are provided as a Source Data file.

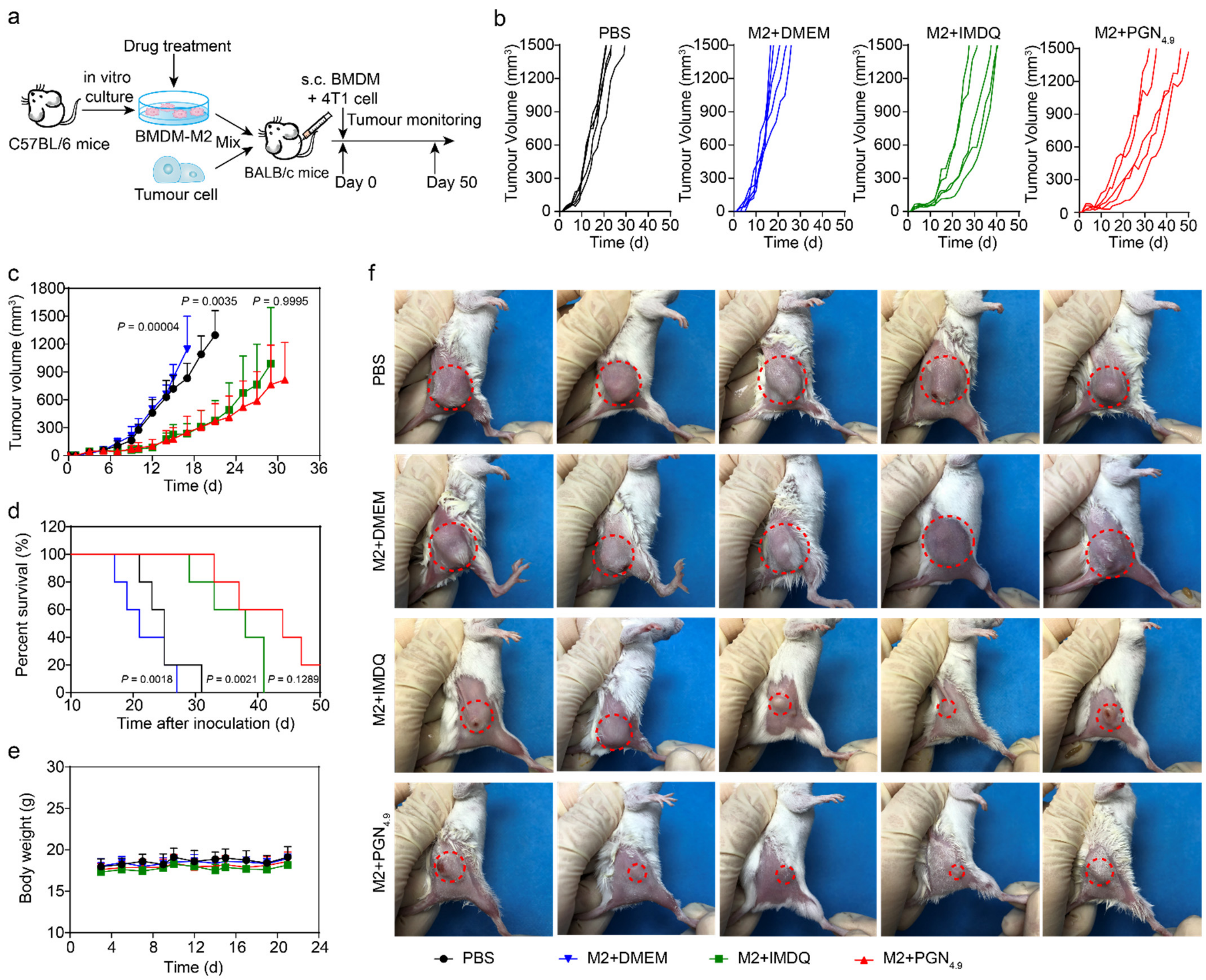

Supplementary Fig. 35. Repolarization of M2-like macrophages to M1 phenotype. (a) Schematic illustration of experiment design for assessing the potential of tumour growth. **(b)** Individual tumour growth kinetics and **(c)** average tumour growth curves of BALB/c mice after subcutaneous co-implantation of 4T1-Luc tumour cells and M2-BMDMs pretreated with DMEM, IMDQ and $PGN_{4.9}$ (equivalent to 2 mg kg^{-1} IMDQ, n = 5 mice per group, two-way ANOVA followed by Tukey's multiple comparisons test). **(d)** Survival curves (n = 5 mice per group; log-rank test; P = 0.0018, P = 0.0021, and P = 0.1289 for M2+$PGN_{4.9}$ versus PBS, M2+DMEM, and M2+IMDQ, respectively). **(e)** Body-weight changes of mice with various groups. **(f)** Photographs of 4T1 tumour-bearing mice on 17 days after tumour inoculation. Source data are provided as a Source Data file.

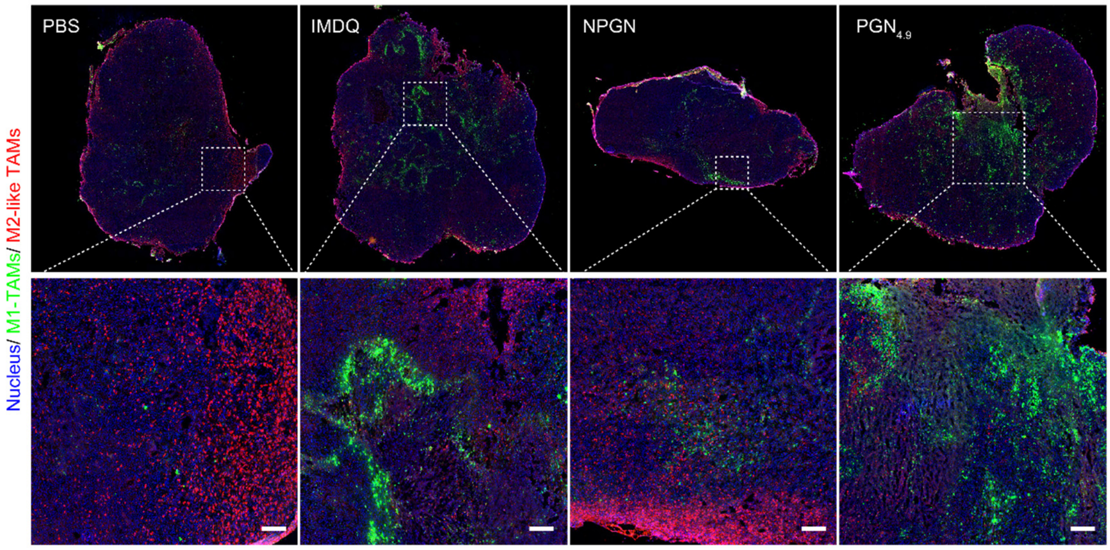

Supplementary Fig. 36. Repolarization of M2-like macrophages to M1 phenotype in vivo. Tumour frozen sections were prepared from the end of 4T1-luc anti-tumour study and stained with iNOS (green) and CD206 (red) in different groups. Whole section (top); magnified part (bottom) (*n* = 3 experiments). Scale bar, 200 μm.

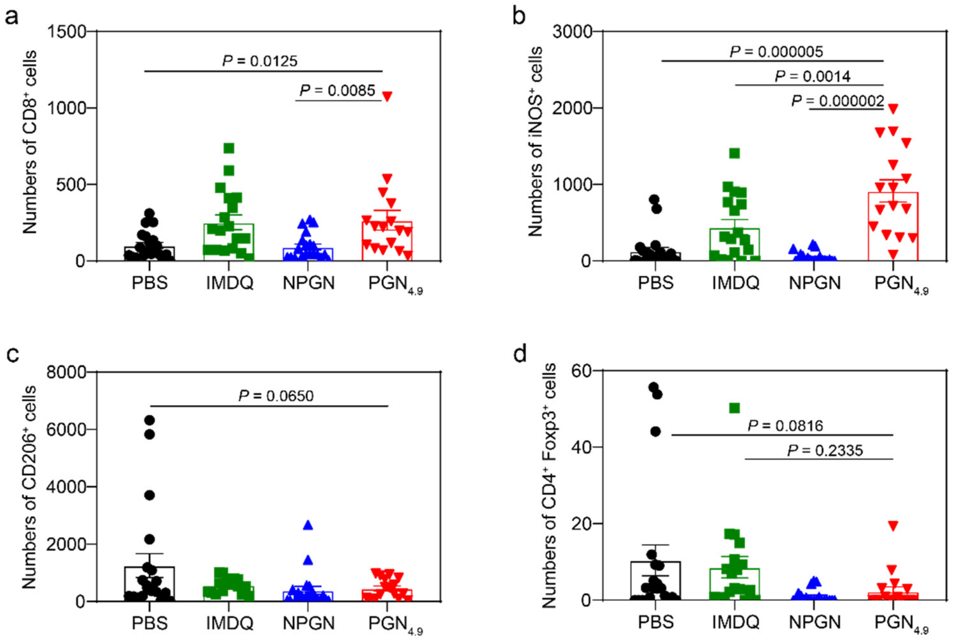

Supplementary Fig. 37. Quantification of tumour infiltration for different immune cells. Cell numbers of **(a)** $CD8^+$ cytotoxic T cells; **(b)** $iNOS^+$ M1-like macrophages; **(c)** $CD206^+$ M2-like macrophages; **(d)** $CD4^+$ $Foxp3^+$ regulatory T cells (*n* = 16 regions for $PGN_{4.9}$ group; *n* = 18 regions for IMDQ group; *n* = 20 regions for other groups, one-way ANOVA followed by Tukey's multiple comparisons test). Source data are provided as a Source Data file.

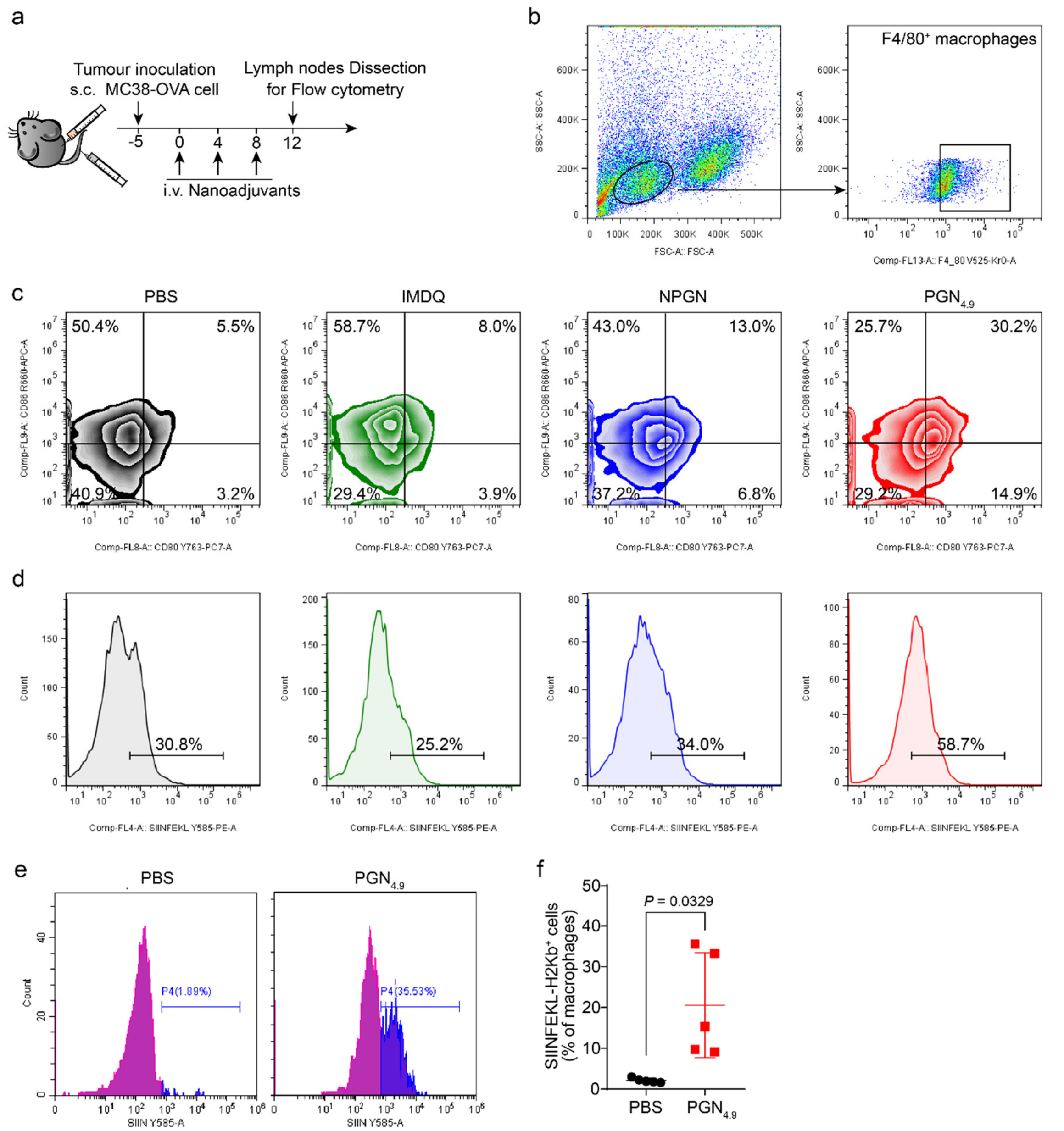

Supplementary Fig. 38. PGN$_{4.9}$ promotes antigen processing and presentation in vivo. (a) Experimental design to evaluate the immunomodulation of PGN$_{4.9}$ in MC38.OVA colorectal model. **(b)** FACS gating strategy for stratification of macrophages in tumour-draining lymph nodes from mice treated different IMDQ formulations (equivalent to 2 mg kg^{-1} IMDQ). **(c)** Representative flow cytometry plots of CD80$^+$CD86$^+$ cells in F4/80$^+$ macrophages. **(d)** Flow cytometry of OVA$_{257\text{-}264}$-positive macrophages in inguinal and popliteal lymph nodes. **(e)** Representative flow cytometry plots of SIINFEKL$^+$ cells in CD11b$^+$F4/80$^+$ macrophages from MC38.OVA tumours. **(f)** Flow cytometry of OVA$_{257\text{-}264}$-positive macrophages in TAMs ($n = 5$ mice per group, two-tailed unpaired Student's t-test). Source data are provided as a Source Data file.

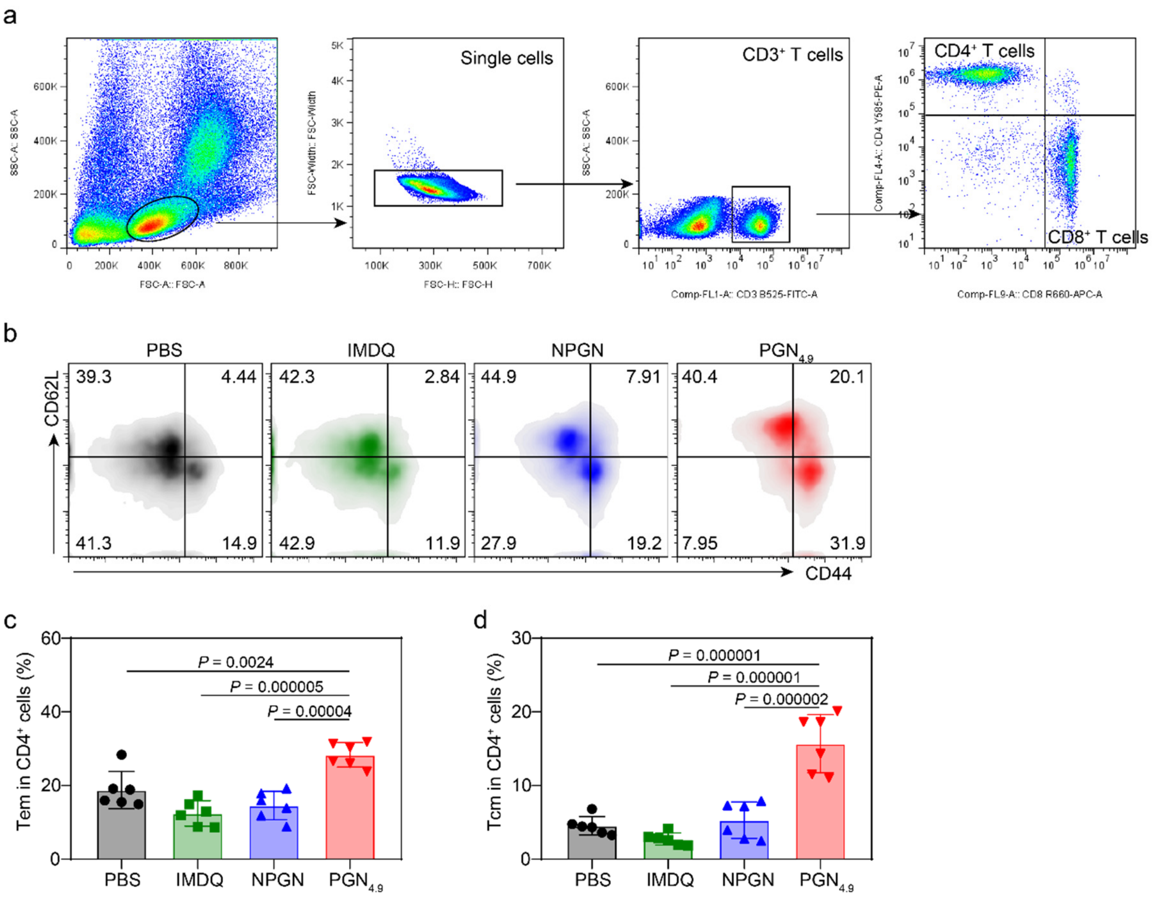

Supplementary Fig. 39. Long-term immune memory. (a) FACS gating strategy for stratification of $CD8^+$ T lymphocytes and $CD4^+$ T lymphocytes from mice **(b)** Representative scatterplots of $CD44^{high}$ $CD62L^{low}$ T cell and $CD44^{high}$ $CD62L^{high}$ T cell subsets among $CD4^+$ T lymphocytes. **(c)** Effector memory T cells and **(d)** central memory T cells of $CD4^+$ T cells from mice upon different treatments ($n = 6$ mice per group, one-way ANOVA followed by Tukey's multiple comparisons test). Source data are provided as a Source Data file.

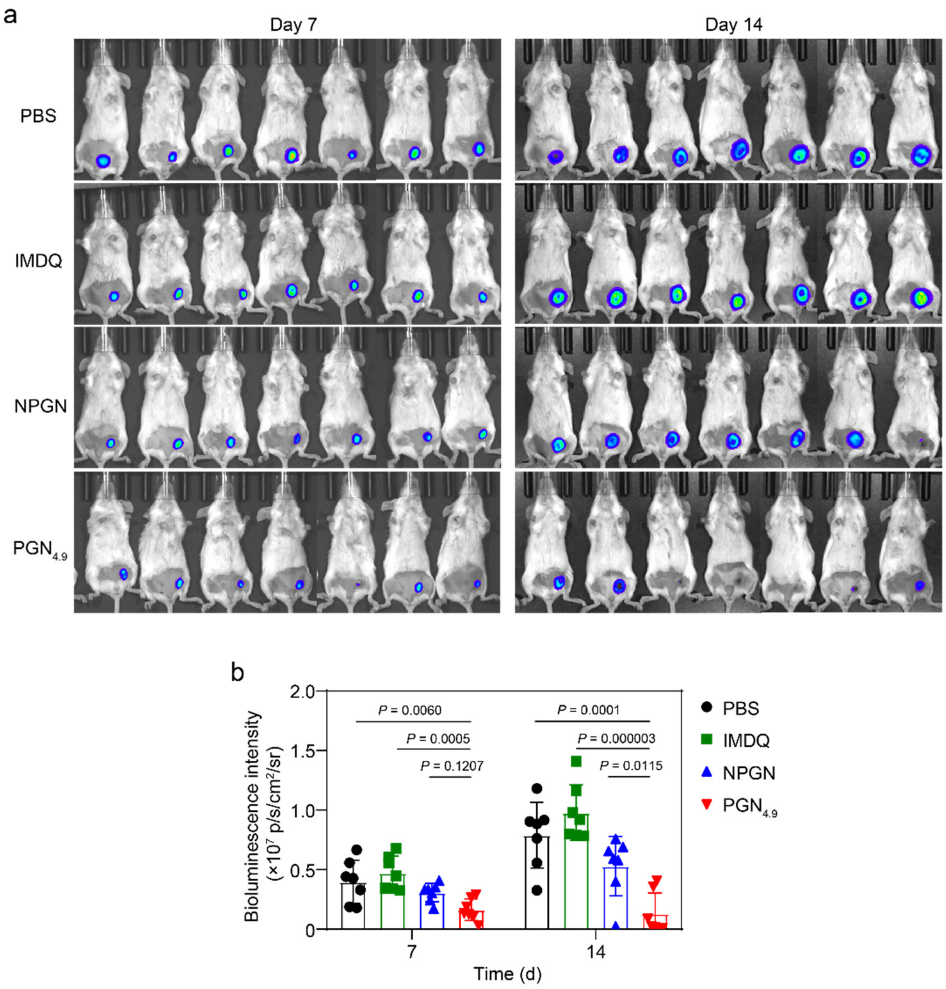

Supplementary Fig. 40. Immunotherapy efficacy on 4T1-luc breast cancer. (a) In vivo IVIS imaging and **(b)** Quantification of bioluminescence intensity in 4T1-luc orthotopic tumour-bearing mice on predesignated days after immunotherapy for three times (n = 7 mice per group, one-way ANOVA followed by Tukey's multiple comparisons test). Source data are provided as a Source Data file.

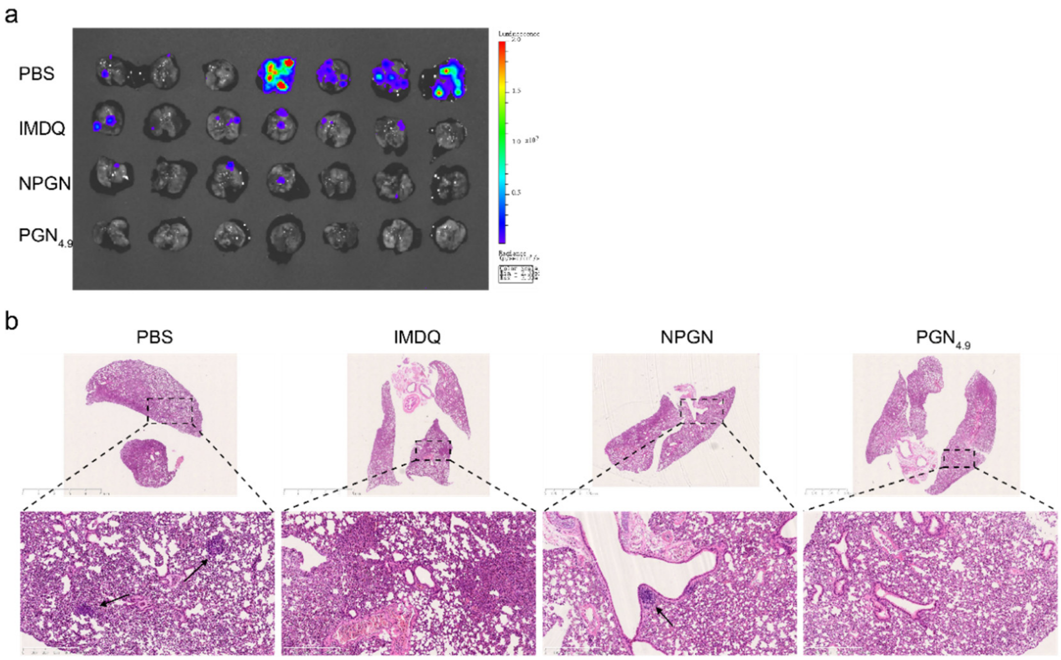

Supplementary Fig. 41. Anti-metastasis study. (a) Bioluminescence images of *ex vivo* lung metastases in 4T1-Luc tumour model upon various IMDQ treatments visualized on day 28 by IVIS Spectrum imaging system (n = 7 mice per group). **(b)** H&E staining of *ex vivo* lungs at the end of immunotherapy (n = 3 experiments). Scale bar, 5 mm.

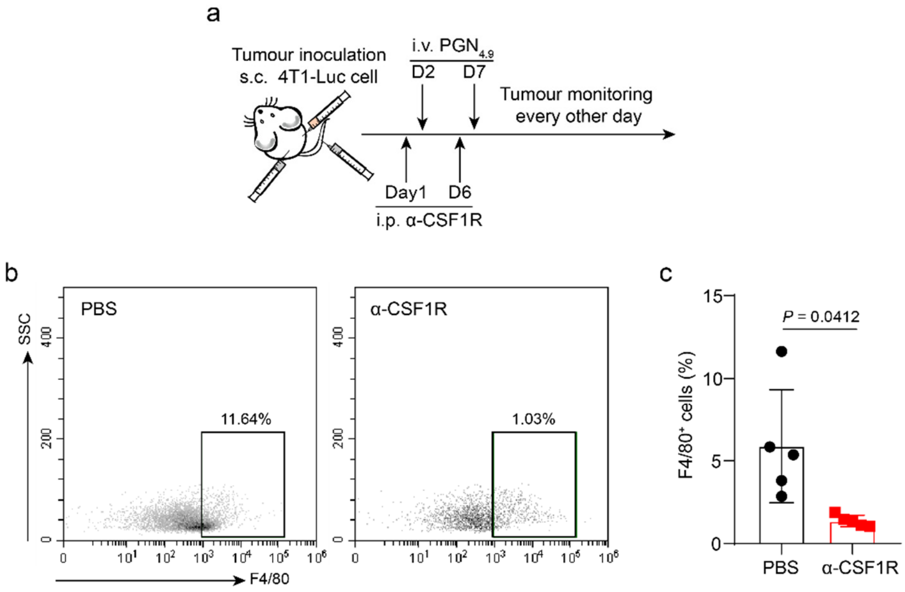

Supplementary Fig. 42. In vivo depletion of macrophages in 4T1 breast tumour model. (a) Schematic illustration for mechanism study of immunotherapy. **(b)** Representative flow cytometry plots of F4/80^{+} macrophages in peripheral blood of mice pretreated with PBS or anti-mouse CSF1R antibody twice. **(c)** Depletion and repopulation of systemic macrophages in 4T1-bearing mice after intraperitoneal injection of α-CSF1R (n = 5 mice per group, two-tailed unpaired Student's t-test). Source data are provided as a Source Data file.

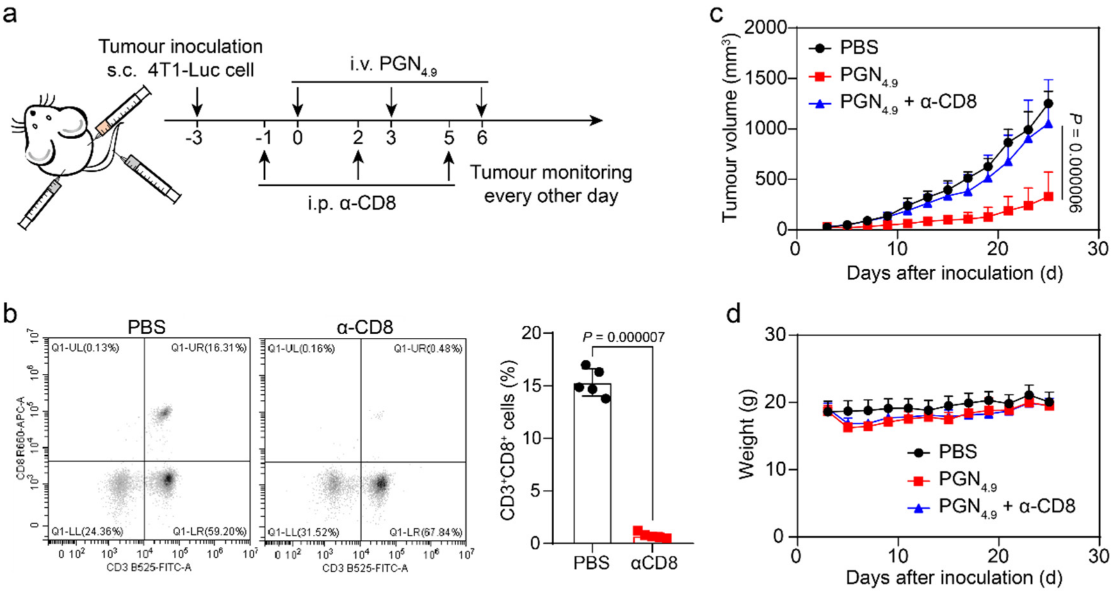

Supplementary Fig. 43. In vivo depletion of T cells in 4T1 breast tumour model. (a) Schematic illustration for mechanism study of immunotherapy. **(b)** Representative flow cytometry plots and repopulation of $CD3^+CD8^+$ cytotoxic T lymphocytes in peripheral blood of mice pretreated with PBS or anti-mouse CD8 antibody three times (*n* = 5 mice per group, two-tailed unpaired Student's t-test). **(c)** Tumour immunotherapy after macrophage depletion in 4T1 tumour-bearing mice (*n* = 7 mice for $PGN_{4.9}$ groups; *n* = 9 mice for other groups, two-way ANOVA followed by Tukey's multiple comparisons test). **(d)** Body-weight changes of mice in various groups (*n* = 7 mice for $PGN_{4.9}$ groups; *n* = 9 mice for other groups). Source data are provided as a Source Data file.

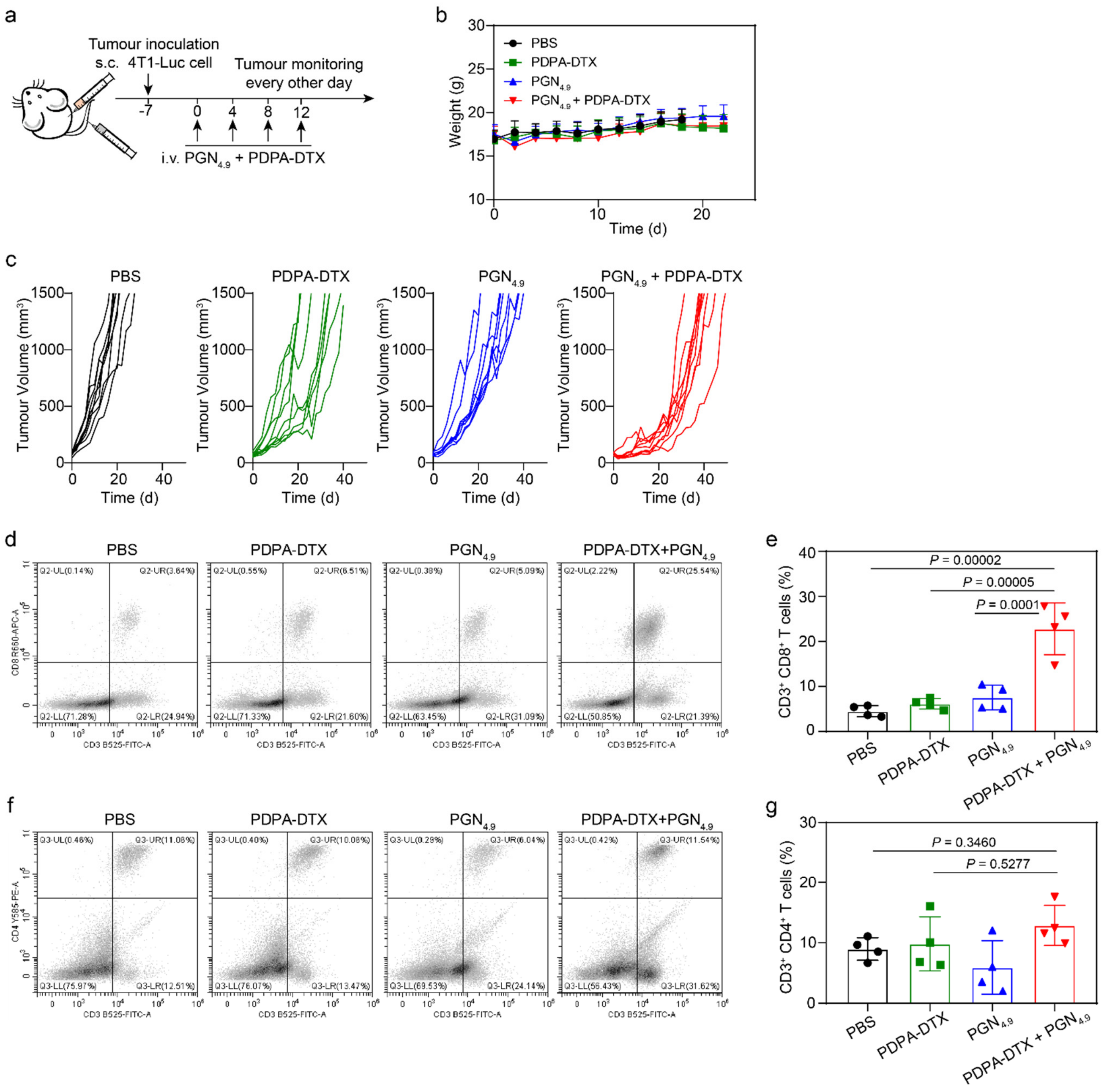

Supplementary Fig. 44. Combined immunotherapy of $PGN_{4.9}$ and PDPA-DTX. (a) Schematic illustration for tumour immunotherapy. **(b)** Body-weight changes of mice treated with PBS, α-PD1, $PGN_{4.9}$ and combined administration. **(c)** Individual tumour growth kinetics of 4T1-luc tumour-bearing mice in different groups (*n* = 9 mice per group). **(d)** Representative flow cytometry plots and **(e)** quantification of $CD3^+CD8^+$ cytotoxic T lymphocytes in MC38 tumours. **(f, g)** The percentage of $CD3^+CD4^+$ T lymphocytes from mice with different treatments (*n* = 4 mice per group, one-way ANOVA followed by Tukey's multiple comparisons test). Source data are provided as a Source Data file.

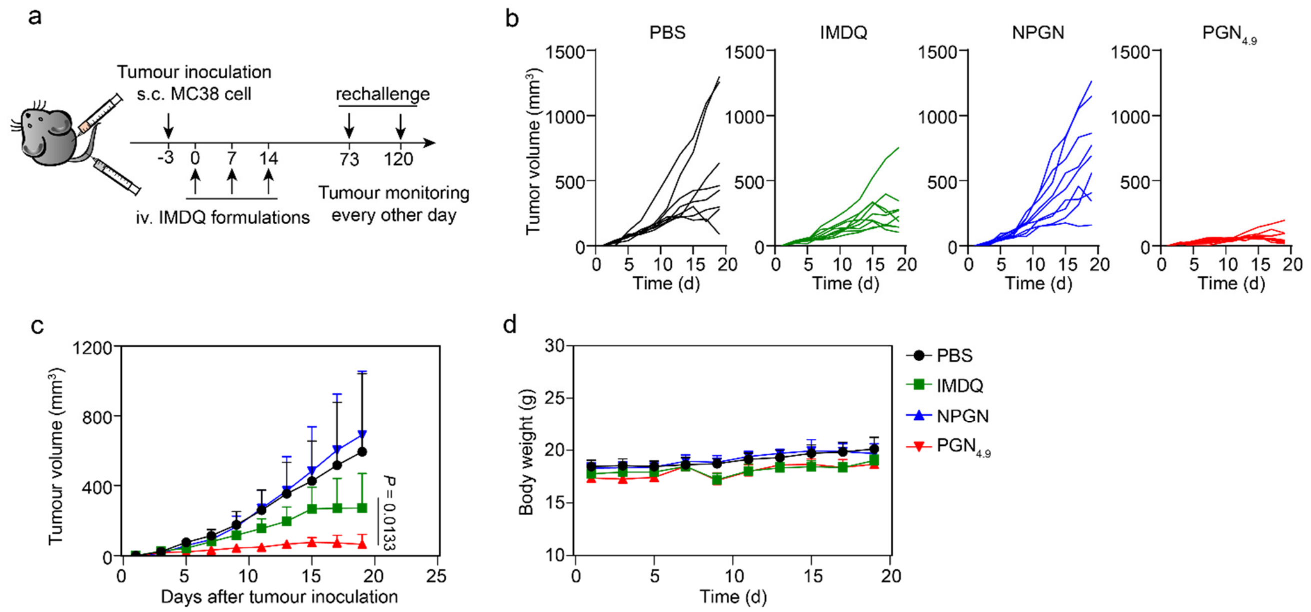

Supplementary Fig. 45. In vivo therapeutic efficacy of PGN4.9 in MC38 colorectal tumour model. (a) Schematic illustration for tumour immunotherapy. **(b)** Individual tumour growth kinetics and **(c)** average tumour growth curves of MC38 tumour-bearing C57BL/6 mice pretreated with PBS, free IMDQ, NPGN and PGN4.9 (*n* = 8 mice for PBS group; *n* = 10 for PGN4.9 group; *n* = 9 mice for other groups, two-way ANOVA followed by Tukey's multiple comparisons test). **(d)** Body-weight changes of mice in various groups. Source data are provided as a Source Data file.

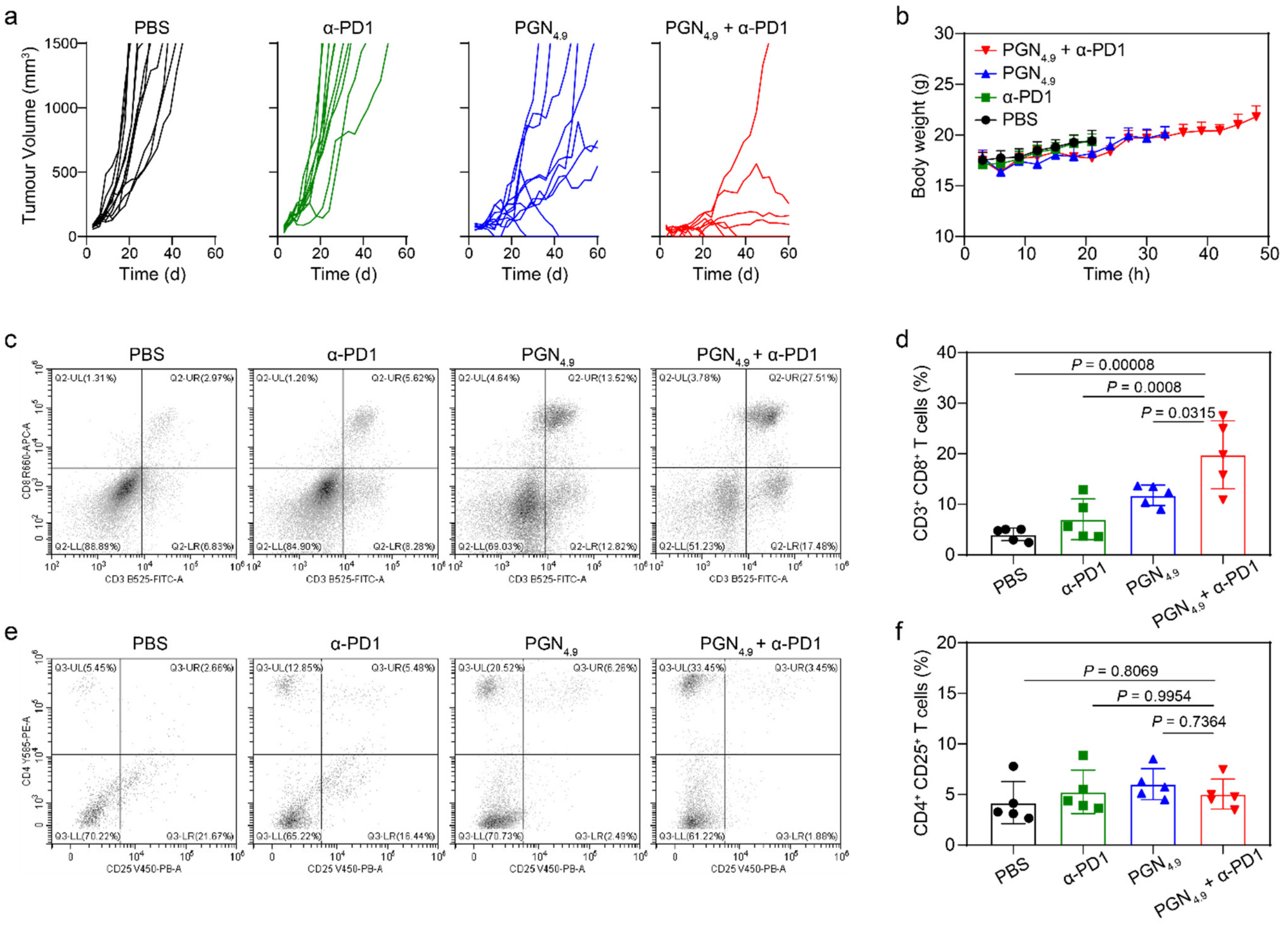

Supplementary Fig. 46. Combined immunotherapy of $PGN_{4.9}$ and α-PD1. (a) Individual tumour growth kinetics of MC38 tumour-bearing mice treated with PBS, α-PD1, $PGN_{4.9}$ and combined administration (*n* = 11 mice for PBS group; *n* = 10 mice for other groups). **(b)** Body-weight changes of mice in different groups. **(c)** Representative flow cytometry plots and **(d)** quantification of $CD3^+CD8^+$ cytotoxic T lymphocytes in MC38 tumours. **(e, f)** The percentage of $CD4^+CD25^+$ regulatory T lymphocytes from mice with different treatments (*n* = 5 mice per group, one-way ANOVA followed by Tukey's multiple comparisons test). Source data are provided as a Source Data file.

2. Supplementary Tables

Supplementary Table 1. Characterization of synthetic copolymers and fluorescent nanoparticles

PGN	Copolymers	pK_a	Dye	pH$_t$	ΔpH$_{10-90\%}$	Dye pairs	R$_F$
**PGN$_{4.5}$**	P(D5A$_{63}$-DBA$_{16}$)	4.49	Cy5	4.46	0.37	BDP FL	69.0
						Cy3.5	1.43
**PGN$_{4.6}$**	P(D5A$_{56}$-DBA$_{24}$)	4.51	Cy5	4.58	0.31	BDP FL	71.6
						Cy3.5	1.35
**PGN$_{4.7}$**	P(D5A$_{50}$-DBA$_{29}$)	4.62	Cy5	4.67	0.27	BDP FL	68.5
						Cy3.5	1.16
**PGN$_{4.8}$**	P(D5A$_{41}$-DBA$_{39}$)	4.72	Cy5	4.75	0.30	BDP FL	77.2
						Cy3.5	1.25
**PGN$_{4.9}$**	P(D5A$_{32}$-DBA$_{47}$)	4.90	Cy5	4.88	0.28	BDP FL	68.4
						Cy3.5	1.25
**PGN$_{5.0}$**	P(D5A$_{22}$-DBA$_{57}$)	4.94	Cy5	4.98	0.25	BDP FL	68.5
						Cy3.5	1.26
**PGN$_{5.1}$**	P(D5A$_{16}$-DBA$_{65}$)	5.06	Cy5	5.09	0.24	BDP FL	63.4
						Cy3.5	1.46
**PGN$_{5.2}$**	P(D5A$_{7}$-DBA$_{72}$)	5.19	Cy5	5.21	0.26	BDP FL	71.9
						Cy3.5	1.23
**PGN$_{5.3}$**	PDBA$_{79}$	5.28	Cy5	5.29	0.25	BDP FL	66.1
						Cy3.5	1.23
**PGN$_{5.4}$**	P(DBA$_{71}$-nDPA$_{8}$)	5.37	Cy5	5.42	0.26	BDP FL	64.1
						Cy3.5	1.28
**PGN$_{5.5}$**	P(DBA$_{62}$-nDPA$_{19}$)	5.47	Cy5	5.51	0.20	BDP FL	81.2
						Cy3.5	1.30

Supplementary Table 2. Characterization of polymer-drug conjugates

copolymers	PEG-P(DBA_{48}-$D5A_{32}$-*r*-IMDQ)	PEG-P(EH_{80}-*r*-IMDQ)
Repeating units of hydrophobic monomer [(i)]	75	82
Drug loading efficiency [(ii)]	5.9%	6.5%
M_n (kDa) [(iii)]	26.8	21.7
M_w (kDa) [(iii)]	29.2	23.2
PDI [(iii)]	1.09	1.07

(i) Repeating units of hydrophobic monomer were measured by ^{1}H-NMR.

(ii) Drug loading efficiency of IMDQ was calculated by UV-Vis spectroscopy.

(iii) M_n, M_w and PDI of copolymers were characterized by gel permeation chromatography.
