# Supplementary material for: pH-gated nanoparticles selectively regulate lysosomal function of tumour-associated macrophages for cancer immunotherapy
Source: Nat Commun. 2023 Sep 21;14:5888. doi: 10.1038/s41467-023-41592-0 (PMC10514266; doi:10.1038/s41467-023-41592-0)
Supplement: Supplementary file 3 — Reporting Summary [file 41467_2023_41592_MOESM3_ESM.pdf]

nature portfolio

Corresponding author(s): Yiguang Wang

Last updated by author(s): Sep 3, 2023

Reporting Summary

Nature Portfolio wishes to improve the reproducibility of the work that we publish. This form provides structure for consistency and transparency in reporting. For further information on Nature Portfolio policies, see our Editorial Policies and the Editorial Policy Checklist.

Statistics

For all statistical analyses, confirm that the following items are present in the figure legend, table legend, main text, or Methods section.

n/a	Confirmed	
☐	☒	The exact sample size (*n*) for each experimental group/condition, given as a discrete number and unit of measurement
☐	☒	A statement on whether measurements were taken from distinct samples or whether the same sample was measured repeatedly
☐	☒	The statistical test(s) used AND whether they are one- or two-sided<br>*Only common tests should be described solely by name; describe more complex techniques in the Methods section.*
☒	☐	A description of all covariates tested
☐	☒	A description of any assumptions or corrections, such as tests of normality and adjustment for multiple comparisons
☐	☒	A full description of the statistical parameters including central tendency (e.g. means) or other basic estimates (e.g. regression coefficient) AND variation (e.g. standard deviation) or associated estimates of uncertainty (e.g. confidence intervals)
☐	☒	For null hypothesis testing, the test statistic (e.g. *F*, *t*, *r*) with confidence intervals, effect sizes, degrees of freedom and *P* value noted<br>*Give P values as exact values whenever suitable.*
☒	☐	For Bayesian analysis, information on the choice of priors and Markov chain Monte Carlo settings
☒	☐	For hierarchical and complex designs, identification of the appropriate level for tests and full reporting of outcomes
☐	☒	Estimates of effect sizes (e.g. Cohen's *d*, Pearson's *r*), indicating how they were calculated

Our web collection on statistics for biologists contains articles on many of the points above.

Software and code

Policy information about availability of computer code

Data collection	ZEN 2010 was used for LSM 880 (Zeiss);<br>NIS-Elements AR 4.20.00 was used for A1R-Storm (Nikon);<br>Living Image 4.3.1 was used for Lumina Series III (PerkinElmer);<br>CytExpert 2.5 was used for CytoFLEX LX (Beckman);<br>MestReNova 9.0 was applied for 1H-NMR;<br>Phenochart 1.0.8 for Vectra-Polaris Automated Quantitative Pathology Imaging System (Perkinelmer).
Data analysis	Statistical analyses were performed on Graphpad Prism 8.0;<br>1H-NMR data was analyzed by MestReNova 9.0;<br>Flow cytometry data were analyzed on FlowJo software package (Flowjo 7.6);<br>Curves were fitted with Origin 2020b;<br>Images were processed with Image-J 1.47 software (NIH).

For manuscripts utilizing custom algorithms or software that are central to the research but not yet described in published literature, software must be made available to editors and reviewers. We strongly encourage code deposition in a community repository (e.g. GitHub). See the Nature Portfolio guidelines for submitting code & software for further information.

Data

Policy information about availability of data

All manuscripts must include a data availability statement. This statement should provide the following information, where applicable:

- Accession codes, unique identifiers, or web links for publicly available datasets
- A description of any restrictions on data availability
- For clinical datasets or third party data, please ensure that the statement adheres to our policy

The source data underlying Figure 2, Figure 3, Figure 4, Figure 5, Figure 6 and western blot are provided with this paper. A reporting summary for this article is available as a Supplementary Information file. The mass spectrometry proteomics data have been deposited to the ProteomeXchange Consortium via the PRIDE partner repository with the dataset identifier PXD044911. Source data are provided with this paper. The remaining data are available within the Article, Supplementary Information or Source Data file.

Research involving human participants, their data, or biological material

Policy information about studies with human participants or human data. See also policy information about sex, gender (identity/presentation), and sexual orientation and race, ethnicity and racism.

Reporting on sex and gender	N/A
Reporting on race, ethnicity, or other socially relevant groupings	N/A
Population characteristics	N/A
Recruitment	N/A
Ethics oversight	N/A

Note that full information on the approval of the study protocol must also be provided in the manuscript.

Field-specific reporting

Please select the one below that is the best fit for your research. If you are not sure, read the appropriate sections before making your selection.

☒ Life sciences ☐ Behavioural & social sciences ☐ Ecological, evolutionary & environmental sciences

For a reference copy of the document with all sections, see nature.com/documents/nr-reporting-summary-flat.pdf

Life sciences study design

All studies must disclose on these points even when the disclosure is negative.

Sample size	Sample size was chosen to assure reproducibility of the experiments in accordance with the replacement, reduction and refinement principles of animal ethics regulation.
Data exclusions	No animals and/or data were excluded.
Replication	All experiments were repeated for at least three times and experimental findings were reproducible. Details of experimental replicates are given in the figure legends.
Randomization	All experimental samples or models including in vitro cells and in vivo mice were randomly allocated to each group.
Blinding	No blinding was performed in this study. The investigators should keep careful track of protocols because that most of the experiments needed multiple treatments (including formulation, cells or mouse tumor treatment, sample collection, and so on). Hence, it would be difficult to blind the investigators to group allocation during data collection and analysis.

Reporting for specific materials, systems and methods

We require information from authors about some types of materials, experimental systems and methods used in many studies. Here, indicate whether each material, system or method listed is relevant to your study. If you are not sure if a list item applies to your research, read the appropriate section before selecting a response.

Materials & experimental systems

n/a	Involved in the study
☐	☒ Antibodies
☐	☒ Eukaryotic cell lines
☒	☐ Palaeontology and archaeology
☐	☒ Animals and other organisms
☒	☐ Clinical data
☒	☐ Dual use research of concern
☒	☐ Plants

Methods

n/a	Involved in the study
☒	☐ ChIP-seq
☐	☒ Flow cytometry
☒	☐ MRI-based neuroimaging

Antibodies

Antibodies used

The anti-Lamp 1 (Cat. No. ab25245, clone number: 1D4B, Dilution 1:1000), anti-iNOS (Cat. No. ab178945, clone number: EPR16635, Dilution 1:1000), anti-liver arginase (Cat. No. ab133543 clone number: EPR6672(B), Dilution 1:2000), anti-cathepsin B (Cat. No. ab214428, clone number: EPR21033, Dilution 1:2000), anti-cathepsin S (ab232740, Dilution 1:1000), anti-mouse F4/80 (ab6640, clone number: CI:A3-1, Dilution 1:500), AF488-conjugated Goat anti-Rabbit (Cat. No. ab150077, Dilution 1:1000), AF488-conjugated Goat anti-Mouse (Cat. No. ab150113, Dilution 1:1000), AF594-cojugated Goat anti-Rat (Cat. No. ab150160, Dilution 1:1000), AF594-cojugated Goat anti-Rabbit (Cat. No. ab150080, Dilution 1:2000), HRP-conjugated goat anti-mouse (Cat. No. ab6789, Dilution 1:10,000) and goat anti-rabbit (Cat. No. ab6721, Dilution 1:10,000), and AF488-conjugated Goat anti-Rat (Cat. No. ab150157, Dilution 1:1000) antibodies were purchased from Abcam.
The anti-Tubulin (Cat. No. T5168, Dilution 1:10,000) and anti-cathepsin L (Cat. No. SAB4300959, Dilution 1:1000) antibodies were purchased from Sigma-Aldrich.
The anti-mouse CD206 (Cat. No. 141702, clone number: C068C2, Dilution 1:200), PerCP/Cy5.5 anti-mouse CD45 (Cat. No. 103132, clone number: 30-F11, Dilution 1:100), FITC anti-mouse/human CD11b (Cat. No. 101205, clone number: M1/70, Dilution 1:200), PE/Cy7 anti-mouse F4/80 (Cat. No. 123114, clone number: BM8, Dilution 1:100), Brilliant Violet 510 Anti-mouse F4/80 (Cat. No. 123135, clone number: BM8, Dilution 1:40), PE-Cy7 anti-mouse CD80 (Cat. No. 104734, clone number: 16-10A1, Dilution 1:40), APC anti-mouse CD86 (Cat. No. 105012, clone number: GL-1, Dilution 1:80), PE anti-mouse CD206 (Cat. No. 141706, clone number: C068C2, Dilution 1:40), PE anti-mouse H-2Kd (Cat. No. 116608, clone number: SF1-1.1, Dilution 1:40), APC anti-mouse CD8α (Cat. No. 100712, clone number: 53-6.7, Dilution 1:80), PE anti-mouse IFN-γ (Cat. No. 505808, clone number: XMG1.2, Dilution 1:100), FITC anti-mouse CD3 (Cat. No. 100204, clone number: 17A2, Dilution 1:50), PE anti-mouse CD4 (Cat. No. 100512, clone number: RM4-5, Dilution 1:80), PE/Cy7 anti-mouse CD44 (Cat. No. 103030, clone number: IM7, Dilution 1:80), Pacific Blue anti-mouse CD62L (Cat. No. 104424, clone number: MEL-14, Dilution 1:200), BV421 anti-mouse CD25 (Cat. No. 102034, clone number: PC61, Dilution 1:200) antibodies were purchased from Biolegend.

Validation

All Antibodies were verified by the supplier and each lot has been quality tested. All validation statements are available on the antibody websites, respectively.
1. Anti-Lamp 1: https://www.abcam.com/lamp1-antibody-1d4b-ab25245.html
2. Anti-iNOS antibody: https://www.abcam.cn/products/primary-antibodies/inos-antibody-epr16635-ab178945.html
3. Anti-liver arginase: https://www.abcam.cn/products/primary-antibodies/liver-arginase-antibody-epr6672b-ab133543.html
4. Anti-cathepsin B: https://www.abcam.cn/products/primary-antibodies/cathepsin-b-antibody-epr21033-ab214428.html
5. Anti-cathepsin S antibody: https://www.abcam.cn/products/primary-antibodies/cathepsin-s-antibody-ab232740.html
6. anti-mouse F4/80 antibody: https://www.abcam.cn/products/primary-antibodies/f480-antibody-cia3-1-macrophage-marker-ab6640.html
7. AF488-conjugated Goat Anti-Rabbit secondary antibody: https://www.abcam.com/goat-rabbit-igg-hl-alexa-fluor-488-ab150077.html
8. AF488-conjugated Goat Anti-Mouse secondary antibody: https://www.abcam.com/goat-mouse-igg-hl-alexa-fluor-488-ab150113.html
9. AF594-cojugated Goat Anti-Rat secondary antibody: https://www.abcam.com/goat-rat-igg-hl-alexa-fluor-594-ab150160.html
10. AF594-cojugated Goat Anti-Rabbit secondary antibody: https://www.abcam.cn/products/secondary-antibodies/goat-rabbit-igg-hl-alexa-fluor-594-ab150080.html
11. HRP-conjugated Goat anti-Rabbit secondary antibody: https://www.abcam.com/goat-rabbit-igg-hl-hrp-ab6721.html
12. HRP-conjugated Goat anti-Mouse secondary antibody: https://www.abcam.cn/products/secondary-antibodies/goat-mouse-igg-hl-hrp-ab6789.html
13. Anti-Tubulin: https://www.sigmaaldrich.com/catalog/product/sigma/t5168?lang=en&region=HK
14. Anti-cathepsin L: https://www.sigmaaldrich.cn/CN/zh/search/sab4300959?focus=products&page=1&perpage=30&sort=relevance&term=sab4300959&type=product
15. Anti-mouse CD206: https://www.biolegend.com/en-us/products/purified-anti-mouse-cd206-mmr-antibody-7317
16. PerCP/Cy5.5 Anti-mouse CD45: https://www.biolegend.com/en-us/products/percp-cyanine5-5-anti-mouse-cd45-antibody-4264
17. FITC Anti-mouse/human CD11b: https://www.biolegend.com/en-us/products/fitc-anti-mouse-human-cd11b-antibody-347
18. PE/Cy7 Anti-mouse F4/80: https://www.biolegend.com/en-us/products/pe-cyanine7-anti-mouse-f4-80-antibody-4070
19. Brilliant Violet 510 Anti-mouse F4/80: https://www.biolegend.com/en-us/products/brilliant-violet-510-anti-mouse-f4-80-antibody-8934
20. PE-Cy7 Anti-mouse CD80: https://www.biolegend.com/en-us/products/pe-cyanine7-anti-mouse-cd80-antibody-9320
21. APC Anti-mouse CD86: https://www.biolegend.com/en-us/products/apc-anti-mouse-cd86-antibody-2896
22. PE Anti-mouse CD206: https://www.biolegend.com/en-us/products/pe-anti-mouse-cd206-mmr-antibody-7424
23. PE Anti-mouse H-2Kd: https://www.biolegend.com/en-us/products/pe-anti-mouse-h-2kd-antibody-1861
24. APC Anti-mouse CD8α: https://www.biolegend.com/en-us/products/apc-anti-mouse-cd8a-antibody-150

25. PE Anti-mouse IFN-γ: https://www.biolegend.com/en-us/products/pe-anti-mouse-ifn-gamma-antibody-997
26. FITC Anti-mouse CD3: https://www.biolegend.com/en-us/products/fitc-anti-mouse-cd3-antibody-45
27. PE Anti-mouse CD4: https://www.biolegend.com/en-us/products/pe-anti-mouse-cd4-antibody-482
28. PE/Cy7 Anti-mouse CD44: https://www.biolegend.com/en-us/products/pe-cyanine7-anti-mouse-human-cd44-antibody-3932
29. Pacific Blue Anti-mouse CD62L: https://www.biolegend.com/en-us/products/pacific-blue-anti-mouse-cd62l-antibody-3117
30. BV421 anti-mouse CD25: https://www.biolegend.com/en-us/products/brilliant-violet-421-anti-mouse-cd25-antibody-7197

Eukaryotic cell lines

Policy information about cell lines and Sex and Gender in Research

Cell line source(s)	4T1 (3101MOUSCSP5056) and MCF-7 breast cancer cells (3101HUMSCSP531), CT26 (1101MOU-PUMC000275), PANC02 pancreatic cancer cell (CRL-2553), RAW264.7 macrophage (TIB-71), NIH/3T3 mouse embryonic fibroblast (1101MOU-PUMC000018), and human umbilical vein endothelial cell (HUVEC, 4201PAT-CCTCC00692) were obtained from National Infrastructure of Cell Line Resource. TLR reporter cell line, RAW-Blue (raw-sp) was purchased from InvivoGen. MC38 colorectal cancer cells and MC38.OVA cells were obtained from Jinming Gao lab. 4T1-GFP cell line was provided by Dr. Yucai Wang (School of Life Sciences, University of Science and Technology of China).
Authentication	None of the cell lines used were authenticated.
Mycoplasma contamination	All cell lines were tested for mycoplasma contamination. No mycoplasma contamination was found.
Commonly misidentified lines (See ICLAC register)	No commonly misidentified cell lines are used in this study.

Animals and other research organisms

Policy information about studies involving animals; ARRIVE guidelines recommended for reporting animal research, and Sex and Gender in Research

Laboratory animals	Female BALB/c (6-8 weeks), C57BL/6 (6-8 weeks), and nu/nu nude mice (6-8 weeks) were sourced from Vital River Laboratory Animal Center (Beijing, China) and maintained in specific pathogen-free (SPF) conditions for one week before the studies. The animals were housed at a temperature of 25 °C in a humidity-controlled environment with free access to food and water in a 12 h light/dark cycle.
Wild animals	No wild animal was used in this study.
Reporting on sex	Female animals were used in this study.
Field-collected samples	No field collected samples were involved in this study.
Ethics oversight	All care and handling of animals were performed with the approval of the Ethics Committee of Peking University (Accreditation number: LA 2019039).

Note that full information on the approval of the study protocol must also be provided in the manuscript.

Flow Cytometry

Plots

Confirm that:

- [x] The axis labels state the marker and fluorochrome used (e.g. CD4-FITC).
- [x] The axis scales are clearly visible. Include numbers along axes only for bottom left plot of group (a 'group' is an analysis of identical markers).
- [x] All plots are contour plots with outliers or pseudocolor plots.
- [x] A numerical value for number of cells or percentage (with statistics) is provided.

Methodology

Sample preparation	Cells were trypsinized, harvested and washed with PBS and then analysed with flow cytometer. In some experiments, cells were stained with antibodies or probes according to the manufacturer's protocols, and then analyzed by flow cytometry.
Instrument	Beckman Coulter CytoFlex Flow Cytometer
Software	FlowJo software package (Flowjo 7.6)
Cell population abundance	Each experiment involved only one type of cell lines and no sorting was performed.

Gating strategy	In general, cells were first gated on FSC/SSC, and single cells were gated using FSC-H and FSC-Width. Cellular uptake, probes and biomarkers were analyzed on the single cell population.

☒ Tick this box to confirm that a figure exemplifying the gating strategy is provided in the Supplementary Information.
